# ﻿Morphological characteristics and phylogenetic analyses reveal five new species of Hymenochaetales (Agaricomycetes, Basidiomycota) from southwestern China

**DOI:** 10.3897/mycokeys.114.143851

**Published:** 2025-02-26

**Authors:** Yunfei Dai, Qi Yuan, Xin Yang, Rui Liu, Defu Liu, Haisheng Yuan, Changlin Zhao

**Affiliations:** 1 College of Forestry, Southwest Forestry University, Kunming 650224, China Southwest Forestry University Kunming China; 2 Kunming Municipal Capital Construction Archives, Kunming 650032, China Kunming Municipal Capital Construction Archives Kunming China; 3 Yunnan Key Laboratory of Gastrodia and Fungal Symbiotic Biology, Zhaotong University, Zhaotong 657000, China Zhaotong University Zhaotong China; 4 Key Laboratory of Forest Ecology and Management, Institute of Applied Ecology, Chinese Academy of Sciences, Shenyang 110016, China Institute of Applied Ecology, Chinese Academy of Sciences Shenyang China

**Keywords:** Biodiversity, Classification, Molecular systematics, New taxa, Wood-inhabiting fungi, Yunnan Province

## Abstract

Wood-inhabiting fungi can decompose wood materials and play a crucial role in the natural world by maintaining the equilibrium of the Earth’s ecosystems. In the present study, five new wood-inhabiting fungal species belonging to the order Hymenochaetales, *Hymenochaeteweishanensis*, *Lyomycesalbofarinaceus*, *Lyomycesalbomarginatus*, *Tubulicrinisalbobadius* and *Xylodonmusicola*, collected from southern China, are proposed based on a combination of morphological features and molecular evidence. *Hymenochaeteweishanensis* is characterized by a coriaceous, tuberculate hymenial surface, a monomitic hyphal system with simple-septate generative hyphae, and ellipsoid to narrow ellipsoid basidiospores (4.0–5.0 × 2.0–3.0 µm); *Lyomycesalbofarinaceus* is characterized by pruinose hymenial surface, a monomitic hyphal system with clamped generative hyphae, and broadly ellipsoid basidiospores (6.0–7.0 × 5.0–6.0 µm); *Lyomycesalbomarginatus* is characterized by the cracked hymenial surface, clamped generative hyphae, and elliposoid basidiospores (4.0–5.5 × 2.7–3.5 µm); *Tubulicrinisalbobadius* is characterized by an arachnoid hymenial surface, a monomitic hyphal system with clamped generative hyphae and cylindrical to allantoid basidiospores (4.0–6.0 × 1.5–2.2 µm) and *Xylodonmusicola* is characterized by an arachnoid hymenial surface, a monomitic hyphal system with clamped generative hyphae and broadly ellipsoid to globe basidiospores (4.0–5.5 × 3.5–5.0 µm). Sequences of the internal transcribed spacers (ITS) and the large subunit (nrLSU) of the nuclear ribosomal DNA (rDNA) markers of the studied samples were generated. Phylogenetic analyses were performed using maximum likelihood, maximum parsimony, and Bayesian inference methods. Full descriptions, illustrations, and phylogenetic analysis results for the five new species are provided.

## ﻿Introduction

Fungi are well-known as a diverse group of microorganisms that play important roles in forest ecosystems (Phookamsak et al. 2019). Mushroom-forming fungi (Agaricomycetes) have the greatest morphological diversity and complexity of fungi (Varga et al. 2019). Wood-inhabiting fungi are essential to natural ecosystems for nutrient cycling and maintaining plant diversity (Drinkwater et al. 2017; Horwath 2017; Hyde et al. 2018; Wu et al. 2022; Guan et al. 2023; Yuan et al. 2023; Deng et al. 2024a, b; Dong et al. 2024; Yuan and Zhao 2024; Zhang et al. 2024). The order Hymenochaetales was described as a monotypic order to accommodate Hymenochaetaceae (Frey et al. 1977; Wu et al. 2022). Hymenochaetales is globally distributed in forest ecosystems, and it comprises 15 families and 84 genera, of which 19 genera have no certain position at the family level (Wu et al. 2022; Wang et al. 2023; Wang and Zhou 2024). Most of the species in Hymenochaetales are polypores and corticioid fungi, which show high morphological diversity and various trophic modes, including saprotrophs, parasites, and symbionts (Wang and Zhou 2024).

The genus *Hymenochaete* Lév. was erected in 1846 and typified by *H.rubiginosa* (Dicks.) Lév. *Hymenochaete* is characterized by annual to perennial, resupinate, effused-reflexed to pileate basidioma with smooth, tuberculate, lamellate, poroid or hydnoid hymenophores; a monomitic or dimitic hyphal system; presence of setae, and hyaline, thin-walled, narrowly cylindrical to globose basidiospores (Léger 1998; Parmasto 2001; He and Dai 2012; Li et al. 2024a). Léger (1998) wrote a worldwide monograph on *Hymenochaete*, providing a key to this genus. According to Index Fungorum (www.indexfungorum.org; accessed on 4 February 2025), the genus *Hymenochaete* has 362 registered names with 235 accepted species worldwide (Léger 1998; Parmasto 2001; Parmasto and Gilbertson 2005; He and Dai 2012; Parmasto 2012; He et al. 2017; Nie et al. 2017; Pacheco et al. 2018; Miettinen et al. 2019; Du et al. 2021a, b; Li et al. 2024a).

The genus *Lyomyces* P. Karst. was introduced by Karsten (1881) and is typified by *L.sambuci* (Pers.) P. Karst. *Lyomyces* comprises corticioid fungi characterized by thin, effused, membranaceous basidiomata that appear fragile in a dry state and show hymenial surface predominantly white or whitish. The hyphal system is monomitic, subicular hyphae thin- or somewhat thick-walled, while the cystidia are thin-walled with tapering, cylindrical, sub-capitate, or capitate apical parts. Basidia are utriform, and the basidiospores are colorless with thin to thick, smooth, or occasionally minutely warted walls (Yurchenko et al. 2024a). The members of *Lyomyces* grow on dead, still-attached, or fallen branches of angiosperms, on dead, wooden, or herbaceous stems, and occasionally on gymnosperm wood (Yurchenko et al. 2017; Chen and Zhao 2020). Molecular studies on *Lyomyces* and related genera have been carried out recently (Riebesehl and Langer 2017; Yurchenko et al. 2017; Viner et al. 2018; Riebesehl et al. 2019; Chen and Zhao 2020; Yuan et al. 2024). Riebesehl and Langer (2017) indicate that *Hyphodontia* s.l. should be divided into several genera as *Hastodontia* (Parmasto) Hjortstam & Ryvarden, *Hyphodontia* J. Erikss, *Kneiffiella* (Pers.) Gray, *Lagarobasidium* Jülich, *Lyomyces* and *Xylodon* (Pers.) Gray and thus thirty-five new combinations were proposed, including fourteen *Lyomyces* species (Dong et al. 2024). The *Lyomycessambuci* complex was clarified based on ITS and 28S sequences analyses and four new species of *Lyomyces* were described (Yurchenko et al. 2017; Dong et al. 2024). Viner et al. (2018) studied the taxonomy of *Lagarobasidium* and *Xylodon* and indicated that twelve species clustered into the *Lyomyces* clade and then grouped with the *Xylodon* clade. Phylogenetic and morphological studies on *Lyomyces* showed that *Lyomyces* grouped with *Hastodontia*, *Hyphodontia*, *Kneiffiella*, and *Xylodon*, in which the *Lyomyces* type species *L.sambuci* was sister to *L.crustosus* (Pers.) P. Karst. formed a single lineage with high support (Riebesehl et al. 2019).

The genus *Tubulicrinis* Donk, typified by *T.glebulosus* (Fr.) Donk (Donk 1956), was a member of the corticioid fungi. They are characterized by resupinate basidiomata, firmly adnate, smooth, pruinose toporulose hymenophore, a monomitic hyphal system with clamped connections on generative hyphae and conspicuous, projecting, amyloid cystidia and small basidia, and cylindrical to allantoid or globose to ellipsoid, thin-walled, smooth, IKI– (both inamyloid and indextrinoid), acyanophilous basidiospores (Donk 1956; Bernicchia and Gorjón 2010; Dong et al. 2024). So far about 46 species have been accepted in the genus worldwide (Donk 1956; Eriksson 1958; Cunningham 1963; Oberwinkler 1966; Hayashi 1974; Ryvarden 1975; Hjortstam 1981; Hjortstam et al. 1988; Rajchenberg 2002; Sharma et al. 2015; Crous et al. 2016; Gruhn et al. 2016; He et al. 2021; Dong et al. 2024). Molecular studies in the genus *Tubulicrinis* have been carried out by Larsson et al. (2006); Dai (2011); Crous et al. (2016); and Dong et al. (2024) and indicated that two *Tubulicrinis* species, *T.gracillimu* (Ellis & Everh. ex D.P. Rogers & H.S. Jacks.) G. Cunn. and *T.subulatus* (Bourdot & Galzin) Donk, formed a monophyletic lineage and then grouped with *Coltricia* clade in Hymenochaetaceae (Larsson et al. 2006). A revised checklist of corticioid and hydnoid fungi showed that six species of *Tubulicrinis* were recorded (Dai 2011); they were nested into the Tubulicrinaceae clade, which belongs to the order Hymenochaetales (Crous et al. 2016; Dong et al. 2024). Based on morphological and molecular analysis of *Tubulicrinis*, two new species were described as *T.xantha* C.L. Zhao and *T.yunnanensis* C.L. Zhao (He et al. 2021).

The genus *Xylodon* (Pers.) Gray is typified by *X.quercinus* (Pers.) Gray (Bernicchia and Gorjón 2010; Yuan and Zhao 2024). The taxa of this genus grow on rotten gymnosperm or angiosperm trunks and stumps, bamboo, and ferns (Greslebin and Rajchenberg 2000; Kotiranta and Saarenoksa 2000; Girometta et al. 2021; Guan et al. 2023; Yuan and Zhao 2024). This genus is characterized by the resupinate or effused basidiomata with a smooth, tuberculate, grandinioid, odontioid, coralloid, irpicoid, or poroid hymenophore; a monomitic or dimitic hyphal system with clamped generative hyphae; the presence of different types of cystidia; utriform or suburniform basidia; and cylindrical to ellipsoid to globose basidiospores (Gray 1821; Bernicchia and Gorjón 2010; Zhang et al. 2024; Yuan and Zhao 2024). Based on the MycoBank database (http://www.mycobank.org, accessed on 4 February 2025 and the Index Fungorum (http://www.indexfungorum.org, accessed on 4 February 2025, 241 specific and infraspecific names are registered for *Xylodon*, of which, 134 are accepted species (Chevallier 1826; Kuntze 1898; Wu 1990, 2000, 2001, 2006; Hjortstam and Ryvarden 2007, 2009; Xiong et al. 2009, 2010; Bernicchia and Gorjón 2010; Tura et al. 2011; Dai 2012; Lee and Langer 2012; Yurchenko and Wu 2014; Zhao et al. 2014; Chen et al. 2016; Kan et al. 2017a, b; Wang and Chen 2017; Viner et al. 2018, 2021; Riebesehl et al. 2019; Shi et al. 2019; Dai et al. 2021; Luo et al. 2021a, 2022; Qu and Zhao 2022; Qu et al. 2022; Guan et al. 2023; Dong et al. 2024; Yuan et al. 2024; Yurchenko et al. 2024b; Zhang et al. 2024).

The present work describes five new species of Hymenochaetales from southwest China, based on the morphology and phylogeny. To clarify the placement and relationships of these new species, we carried out a phylogenetic and taxonomic study based on the combined ITS+nLSU and ITS only sequences analyses. Full descriptions, illustrations, and comparison of five new species with closely related taxa and phylogenetic trees showing the placement of five new species within the order Hymenochaetales are provided.

## ﻿Materials and methods

### ﻿Sample collection and herbarium specimen preparation

The fresh fruiting bodies were collected on the fallen angiosperm branches which came from Dali, Zhaotong, and Qujing of Yunnan Province, China, and the important collection information was noted (Rathnayaka et al. 2024). The samples were photographed in situ, and fresh macroscopic details were recorded. Photographs were recorded by a Nikon D7100 camera. All the photos were focus-stacked using Helicon Focus software, and macroscopic details were recorded. Specimens were dried in an electric food dehydrator at 40 °C (Hu et al. 2022; Dong et al. 2024). Once dried, the specimens were sealed in an envelope and zip-lock plastic bags and labeled (Dong et al. 2024). The dried specimens were deposited in the herbarium of the Southwest Forestry University (SWFC), Kunming, Yunnan Province, China.

### ﻿Morphology

The macromorphological descriptions were based on field notes and photos captured in the field and lab. The color terminology follows Petersen (1996). The micromorphological data were obtained from the dried specimens after observation under a light microscope with a magnification of 10 × 100 (Zhao et al. 2023; Dong et al. 2024). Sections were mounted in 5% KOH and 2% phloxine B dye (C_20_H_2_Br_4_C_l4_Na_2_O_5_), and we also used other reagents, including Cotton Blue and Melzer’s reagent to observe micromorphology following (Dong et al. 2024). To show the variation in spore sizes, 5% of measurements were excluded from each end of the range and shown in parentheses. At least thirty basidiospores from each specimen were measured. Stalks were excluded from basidia measurements, and the hilar appendage was excluded from basidiospores measurements. The following abbreviations are used: KOH = 5% potassium hydroxide water solution, CB– = acyanophilous, IKI– = both inamyloid and non-dextrinoid, L = mean spore length (arithmetic average for all spores), W = mean spore width (arithmetic average for all spores), Q = variation in the L/W ratios between the specimens studied, and n = a/b (number of spores (a) measured from given number (b) of specimens).

### ﻿Molecular phylogeny

The CTAB rapid plant genome extraction kit-DN14 (Aidlab Biotechnologies Co., Ltd., Beijing, China) was used to obtain genomic DNA from the dried specimens according to the manufacturer’s instructions. The ITS region was amplified with ITS5 and ITS4 primers (White et al. 1990). The nLSU region was amplified with the LR0R and LR7 (Vilgalys and Hester 1990; Rehner and Samuels 1994). The PCR procedure for ITS was as follows: initial denaturation at 95 °C for 3 min, followed by 35 cycles at 94 °C for 40 s, 58 °C for 45 s, and 72 °C for 1 min, and a final extension of 72 °C for 10 min. The PCR procedure for nLSU was as follows: initial denaturation at 94 °C for 1 min, followed by 35 cycles at 94 °C for 30 s, 48 °C for 1 min, and 72 °C for 1.5 min, and a final extension of 72 °C for 10 min. The PCR products were purified and sequenced at Kunming Tsingke Biological Technology Limited Company (Yunnan Province, P.R. China). The newly generated sequences were deposited in NCBI GenBank (Table 1).

**Figure 1.**
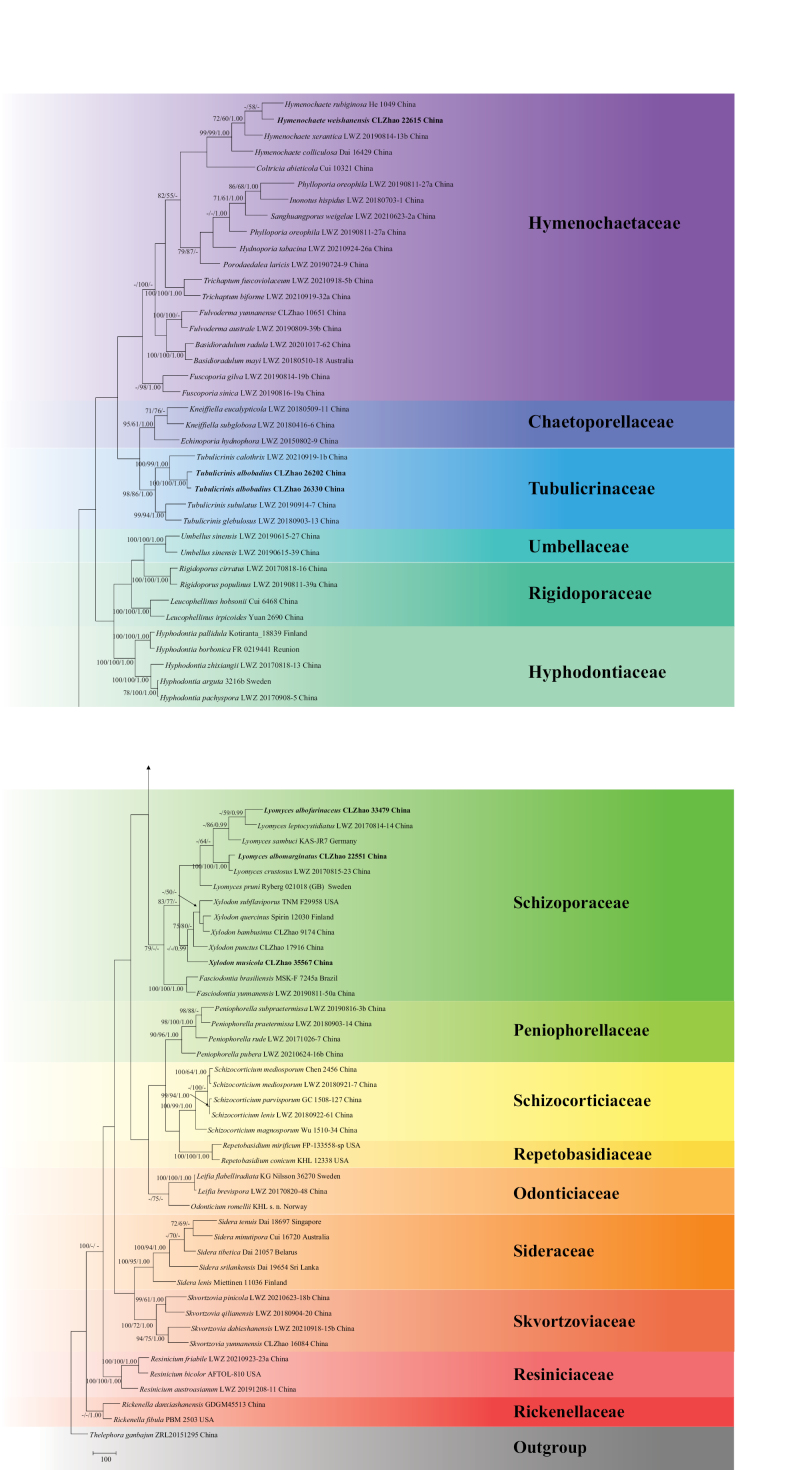
Maximum parsimony strict consensus tree illustrating the phylogeny of the order Hymenochaetales based on ITS+nLSU sequences. Branches are labelled with maximum likelihood bootstrap value ≥ 70%, parsimony bootstrap value ≥ 50%, and Bayesian posterior probabilities ≥ 0.95.

**Figure 2.**
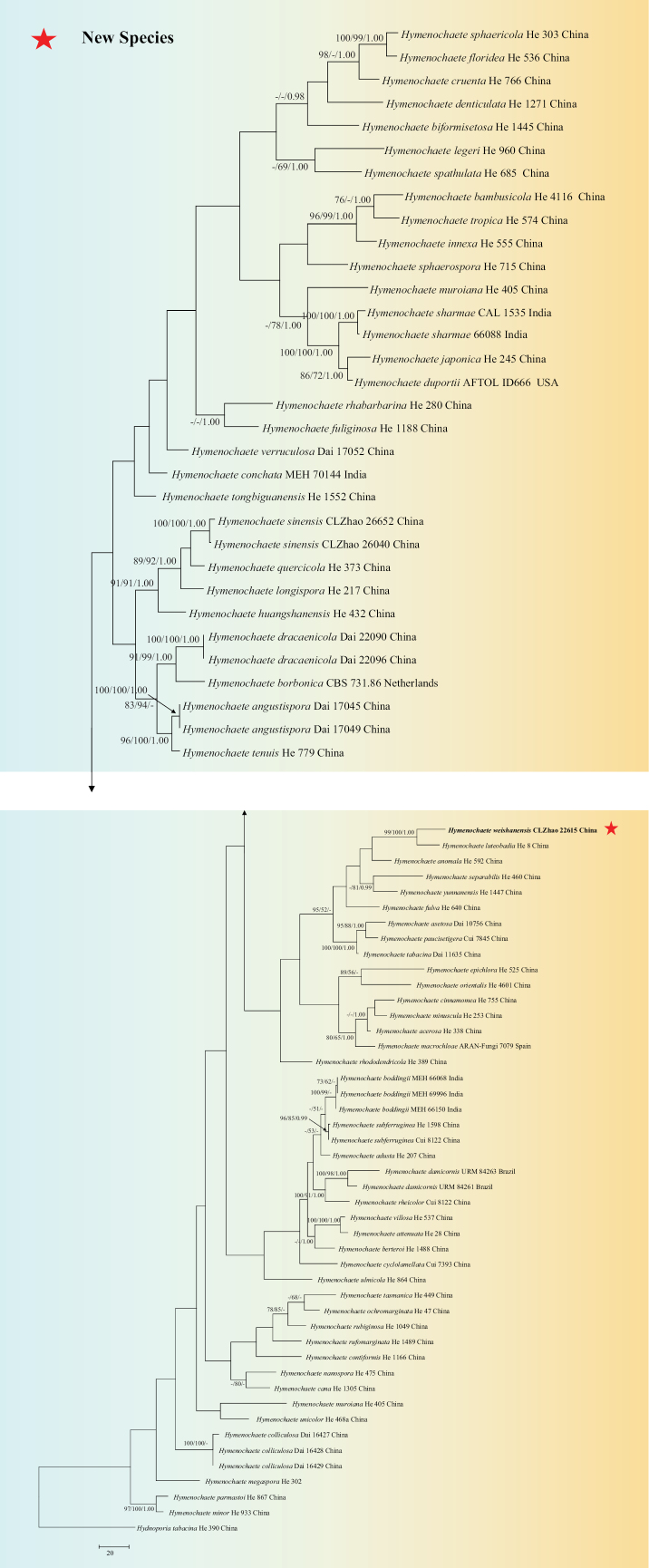
Maximum parsimony strict consensus tree illustrating the phylogeny of the one new species and related species in the genus *Hymenochaete* based on ITS sequences. Branches are labelled with maximum likelihood bootstrap value ≥ 70%, parsimony bootstrap value ≥ 50%, and Bayesian posterior probabilities ≥ 0.95.

**Figure 3.**
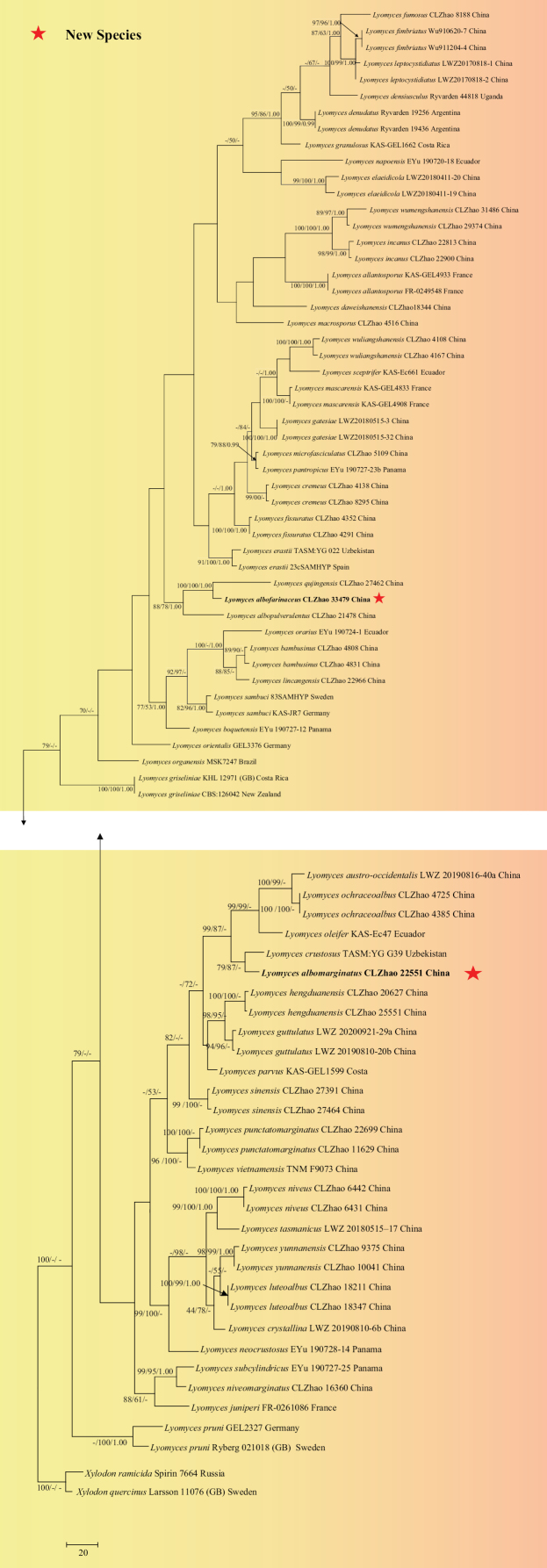
Maximum parsimony strict consensus tree illustrating the phylogeny of the new species and related species in the genus *Lyomyces* based on ITS sequences. Branches are labelled with maximum likelihood bootstrap value ≥ 70%, parsimony bootstrap value ≥ 50%, and Bayesian posterior probabilities ≥ 0.95.

**Figure 4.**
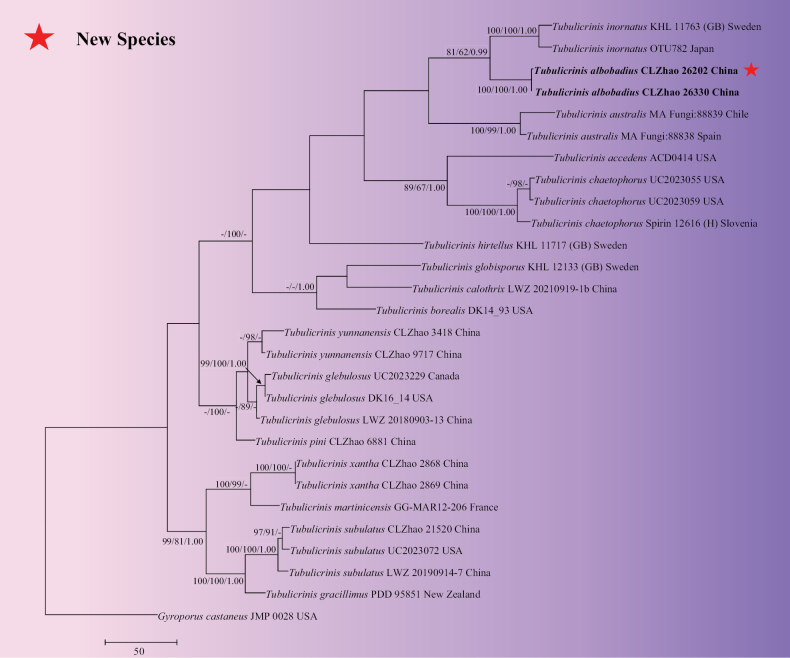
Maximum parsimony strict consensus tree illustrating the phylogeny of the new species and related species in the genus *Tubulicrinis* based on ITS sequences. Branches are labelled with maximum likelihood bootstrap value ≥ 70%, parsimony bootstrap value ≥ 50%, and Bayesian posterior probabilities ≥ 0.95.

**Figure 5.**
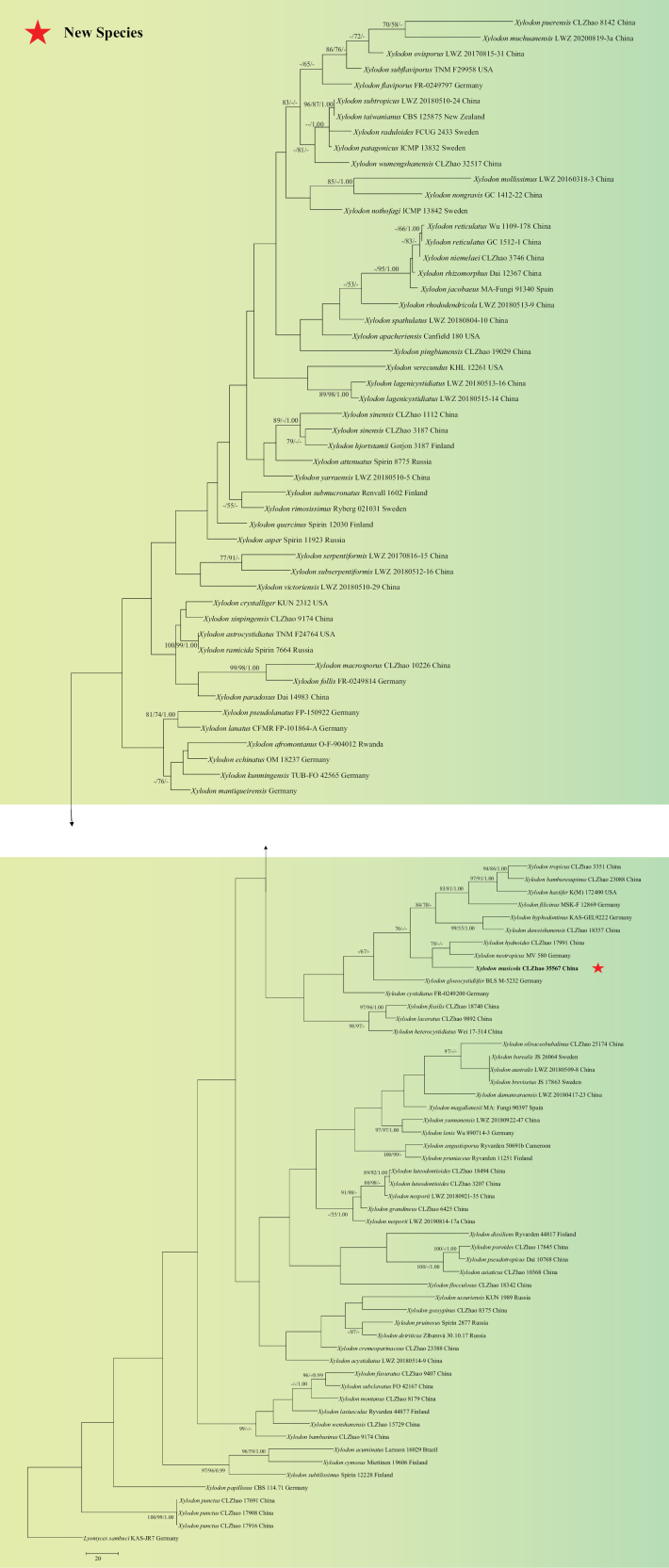
Maximum parsimony strict consensus tree illustrating the phylogeny of the new species and related species in the genus *Xylodon* based on ITS sequences. Branches are labelled with maximum likelihood bootstrap value ≥ 70%, parsimony bootstrap value ≥ 50%, and Bayesian posterior probabilities ≥ 0.95.

The sequences were aligned in MAFFT version 7 (Katoh et al. 2019) using the G-INS-i strategy. The alignment was adjusted manually using AliView version 1.27 (Larsson 2014). A sequence of *Thelephoraganbajun* obtained from GenBank was used as an outgroup to root trees in the ITS+nLSU analysis (Fig. 1) in the order Hymenochaetales (Wang and Zhou 2024) (TreeBASE WEB submission ID 31958). Sequence of *Hydnoporiatabacina* (Sowerby) Spirin, Miettinen & K.H. Larss. obtained from GenBank was used as an outgroup to root trees in the ITS analysis in the genus *Hymenochaete* (Fig. 2) (TreeBASE WEB submission ID 31959). Sequences of *Xylodonquercinus* (Pers.) Gray and *Xylodonramicida* Spirin & Miettinen obtained from GenBank were used as outgroups to root trees in the ITS analysis in the genus *Lyomyces* (Fig. 3) (TreeBASE WEB submission ID 31960). A sequence of *Gyroporuscastaneus* (Bull.) Quél. obtained from GenBank was used as an outgroup to root trees in the ITS analysis in the genus *Tubulicrinis* (Fig. 4) (TreeBASE WEB submission ID 31961). A sequence of *Lyomycessambuci* (Pers.) P. Karst. obtained from GenBank was used as an outgroup to root trees in the ITS analysis in the genus *Xylodon* (Fig. 5) (TreeBASE WEB submission ID 31962).

**Table 1.** List of species, specimens, and GenBank accession number of sequences used in this study. [New species is shown in bold; * type material; – is shown data without used].

Order/Family	Species Name	Sample No.	GenBank Accession No.			References
			ITS	nLSU		
Boletales/Gyroporaceae	*Gyroporuscastaneus*	JMP 0028	EU819468	–	USA	Palmer et al. (2008)
Hymenochaetales/ Chaetoporellaceae	*Echinoporiahydnophora*	LWZ 20150802-9	ON063639	ON063838	China	Wang et al. (2023)
	*Kneiffiellaeucalypticola*	LWZ 20180509-11	MT319410	MT319142	China	Wang et al. (2021b)
	*Kneiffiellasubglobosa*	LWZ 20180416-6	MT319413	MT319145	China	Wang et al. (2021b)
-/Hymenochaetaceae	*Basidioradulummayi*	LWZ 20180510-18	MN017785	MN017792	Australia	Wang et al. (2021a)
	*Basidioradulumradula*	LWZ 20201017-62	ON063684	ON063884	China	Wang et al. (2023)
	*Coltriciaabieticola*	Cui 10321	KX364785	KX364804	China	Bian and Dai (2017)
	*Fulvodermaaustrale*	LWZ 20190809-39b	ON063644	ON063843	China	Wang et al. (2023)
	*Fulvodermayunnanense*	CLZhao 10651	OL619278	OL619278	China	Direct Submission
	*Fuscoporiagilva*	MSU653	JF461327	JF461327	Thailand	Insumran et al. (2012)
	*Fuscoporiasinica*	LWZ 20190816-19a	ON063649	ON427358	China	Wang et al. (2023)
	*Hydnoporiatabacina*	LWZ 20210924-26a	ON063651	ON063851	China	Wang et al. (2023)
	*Hydnoporiatabacina*	He 390	JQ279610	–	China	He et al.(2017)
	*Hymenochaeteacerosa*	He 338	JQ279543	–	China	He et al.(2017)
	*Hymenochaeteadusta*	He 207	JQ279523	–	China	He et al.(2017)
	*Hymenochaeteangustispora*	Dai 17045	MF370592	–	China	He et al.(2017)
	*Hymenochaeteangustispora*	Dai 17049	MF370593	–	China	He et al.(2017)
	*Hymenochaeteanomala*	He 592	JQ279566	–	China	He et al.(2017)
	*Hymenochaeteasetosa*	Dai 10756	JQ279559	–	China	He et al.(2017)
	*Hymenochaeteattenuata*	He 28	JQ279526	–	China	He et al.(2017)
	*Hymenochaetebambusicola*	He 4116	KY425674	–	China	He et al.(2017)
	*Hymenochaeteberteroi*	He 1488	KU975459	–	China	He et al.(2017)
	*Hymenochaetebiformisetosa*	He 1445	KF908247	–	China	Yang and He (2014)
	*Hymenochaeteboddingii*	MEH 66068	MN030343	–	India	Du et al.(2021a)
	*Hymenochaeteboddingii*	MEH 69996	MN030341	–	India	Du et al.(2021a)
	*Hymenochaeteboddingii*	MEH 66150	MN030344	–	India	Du et al.(2021a)
	*Hymenochaeteborbonica*	CBS 731.86	MH862026	–	Netherlands	Du et al.(2021a)
	*Hymenochaetecana*	He 1305	KF438169	–	China	He et al.(2017)
	*Hymenochaetecinnamomea*	He 755	JQ279548	–	China	He et al.(2017)
	*Hymenochaetecolliculosa*	Dai 16427	MF370595	–	China	He et al.(2017)
	*Hymenochaetecolliculosa*	Dai 16428	MF370596	–	China	He et al.(2017)
	*Hymenochaetecolliculosa*	Dai 16429	MF370597	–	China	He et al.(2017)
	*Hymenochaeteconchata*	MEH 70144	MF373838	–	India	Du et al.(2021a)
	*Hymenochaetecontiformis*	He 1166	KU975461	–	China	He et al.(2017)
	*Hymenochaetecruenta*	He 766	JQ279595	–	China	He et al.(2017)
	*Hymenochaetecyclolamellata*	Cui 7393	JQ279513	–	China	He et al.(2017)
	*Hymenochaetedamicornis*	URM 84261	KC348466	–	Brazil	Du et al.(2021a)
	*Hymenochaetedamicornis*	URM 84263	KC348467	–	Brazil	Du et al.(2021a)
	*Hymenochaetedenticulata*	He 1271	KF438171	–	China	He et al. (2017)
	*Hymenochaetedracaenicola*	Dai 22090	MW559797	–	China	Du et al. (2021a)
	*Hymenochaetedracaenicola*	Dai 22096	MW559798	–	China	Du et al. (2021a)
	*Hymenochaeteduportii*	AFTOL ID666	DQ404386	–	USA	He et al.(2017)
-/Hymenochaetaceae	*Hymenochaeteepichlora*	He 525	JQ279549	–	China	He and Dai (2012)
	*Hymenochaetefloridea*	He 536	JQ279597	–	China	He and Dai (2012)
	*Hymenochaetefuliginosa*	He 1188	KU975465	–	China	Du et al.(2021a)
	*Hymenochaetefulva*	He 640	JQ279565	–	China	He and Dai (2012)
	*Hymenochaetehuangshanensis*	He 432	JQ279533	–	China	He and Dai (2012)
	*Hymenochaetejaponica*	He 245	JQ279590	–	China	He and Dai (2012)
	*Hymenochaeteinnexa*	He 555	JQ279584	–	China	He and Dai (2012)
	*Hymenochaetelegeri*	He 960	KU975469	–	China	He et al.(2017)
	*Hymenochaetelongispora*	He 217	JQ279537	–	China	He and Dai (2012)
	*Hymenochaeteluteobadia*	He 8	JQ279569	–	China	He and Dai (2012)
	*Hymenochaetemacrochloae*	ARAN-Fungi 7079	MF990738	–	Spain	Du et al.(2021a)
	*Hymenochaetemegaspora*	He 302	JQ279553	–	China	He and Dai (2012)
	*Hymenochaeteminor*	He 933	JQ279555	–	China	He and Dai (2012)
	*Hymenochaeteminuscula*	He 253	JQ279546	–	China	He and Dai (2012)
	*Hymenochaetemuroiana*	He 405	JQ279542	–	China	Du et al.(2021a)
	*Hymenochaetenanospora*	He 475	JQ279531	–	China	He and Dai (2012)
	*Hymenochaeteochromarginata*	He 47	JQ279579	–	China	He and Dai (2012)
	*Hymenochaetetabacina*	Dai 11635	JQ279563	–	China	He and Dai (2012)
	*Hymenochaeteorientalis*	He 4601	KY425677	–	China	He et al.(2017)
	*Hymenochaeteparmastoi*	He 867	JQ780063	–	China	He et al.(2017)
	*Hymenochaetepaucisetigera*	Cui 7845	JQ279560	–	China	He and Dai (2012)
	*Hymenochaetequercicola*	He 373	KU975474	–	China	He et al.(2017)
	*Hymenochaeterhabarbarina*	He 280	JQ279574	–	China	He and Dai (2012)
	*Hymenochaeterheicolor*	Cui 8317	JQ279529	–	China	Du et al.(2021a)
	*Hymenochaeterhododendricola*	He 389	JQ279577	–	China	He and Dai (2012)
	*Hymenochaeterubiginosa*	He 1049	JQ716407	–	China	Yang et al.(2016)
	*Hymenochaeterufomarginata*	He 1489	KU975477	–	China	He et al.(2017)
	*Hymenochaetesharmae*	CAL 1535	KY929017	–	India	Du et al.(2021a)
	*Hymenochaetesharmae*	66088	MK588753	–	India	Du et al.(2021a)
	*Hymenochaetesinensis*	CLZhao 26040	OR659001	–	China	Li et al. (2024a)
	*Hymenochaetesinensis*	CLZhao 26652	PQ060540	–	China	Li et al. (2024a)
	*Hymenochaeteseparabilis*	He 460	JQ279572	–	China	He and Dai (2012)
	*Hymenochaetespathulata*	He 685	JQ279591	–	China	He et al.(2017)
	*Hymenochaetesphaericola*	He 303	JQ279599	–	China	He and Dai (2012)
	*Hymenochaetesphaerospora*	He 715	JQ279594	–	China	He et al.(2017)
	*Hymenochaetesubferruginea*	Cui 8122	JQ279521	–	China	Du et al.(2021a)
	*Hymenochaetesubferruginea*	He 1598	KU975481	–	China	Du et al.(2021a)
	*Hymenochaetetasmanica*	He 449	JQ279582	–	China	He et al.(2017)
	*Hymenochaetetenuis*	He 779	JQ279538	–	China	Du et al.(2021a)
	*Hymenochaetetongbiguanensis*	He 1552	KF908248	–	China	He et al.(2017)
	*Hymenochaetetropica*	He 574	JQ279587	–	China	He et al.(2017)
	*Hymenochaeteulmicola*	He 864	JQ780065	–	China	He et al.(2017)
	*Hymenochaeteunicolor*	He 468a	JQ279551	–	China	He et al.(2017)
-/Hymenochaetaceae	*Hymenochaeteverruculosa*	Dai 17052	MF370594	–	China	He et al.(2017)
	*Hymenochaetevillosa*	He 537	JQ279528	–	China	He et al.(2017)
	***Hymenochaeteweishanensis***	CLZhao 22615*	**PQ523357**	**PQ523363**	**China**	**Present study**
	*Hymenochaetexerantica*	LWZ 20190814-13b	ON063657	ON063856	China	Wang et al. (2023)
	*Hymenochaeteyunnanensis*	He 1447	KU975486	–	China	He et al.(2017)
	*Inonotushispidus*	LWZ 20180703-1	ON063659	ON063858	China	Wang et al. (2023)
	*Phellinuspiceicola*	LWZ 20190921-5	ON063662	ON063862	China	Wang et al. (2023)
	*Phylloporiaoreophila*	LWZ 20190811-27a	ON063665	ON063865	China	Wang et al. (2023)
	*Porodaedalealaricis*	LWZ 20190724-9	ON063668	ON063868	China	Wang et al. (2023)
	*Sanghuangporusweigelae*	LWZ 20210623-2a	ON063671	ON063870	China	Wang et al. (2023)
	*Trichaptumbiforme*	LWZ 20210919-32a	ON063701	ON063901	China	Wang et al. (2023)
	*Trichaptumfuscoviolaceum*	LWZ 20210918-5b	ON063703	ON063903	China	Wang et al. (2023)
	*Hyphodontiaarguta*	3216b	DQ873605	DQ873605	Sweden	Larsson et al. (2006)
	*Hyphodontiaborbonica*	FR 0219441	KR349240	KR349240	Reunion	Riebesehl and Langer (2017)
	*Hyphodontiapachyspora*	LWZ 20170908-5	MT319426	MT319160	China	Wang et al. (2021b)
	*Hyphodontiapallidula*	Kotiranta_18839	OP620785	OP620785	Finland	Viner et al. (2023)
	*Hyphodontiazhixiangii*	LWZ 20170818-13	MT319420	MT319151	China	Wang et al. (2021b)
-/Odonticiaceae	*Leifiabrevispora*	LWZ 20170820-48	MK343470	MK343474	China	Liu et al. (2019)
	*Leifiaflabelliradiata*	KG Nilsson 36270	DQ873635	DQ873635	Sweden	Larsson et al. (2006)
	*Odonticiumromellii*	KHL s. n.	DQ873639	DQ873639	Norway	Larsson et al. (2006)
-/Peniophorellaceae	*Peniophorellapraetermissa*	LWZ 20180903-14	ON063686	ON063886	China	Wang et al. (2023)
	*Peniophorellapubera*	LWZ 20210624-16b	ON063687	ON063887	China	Wang et al. (2023)
	*Peniophorellarude*	LWZ 20171026-7	ON063688	ON063888	China	Wang et al. (2023)
	*Peniophorellasubpraetermissa*	LWZ 20190816-3b	ON063689	ON063889	China	Wang et al. (2023)
-/Repetobasidiaceae	*Repetobasidiumconicum*	KHL 12338	DQ873647	DQ873647	USA	Larsson et al. (2006)
	*Repetobasidiummirificum*	FP-133558-sp	–	AY293208	USA	Binder et al. (2005)
-/Resiniciaceae	*Resiniciumaustroasianum*	LWZ 20191208-11	ON063691	ON063891	China	Wang et al. (2023)
	*Resiniciumbicolor*	AFTOL-810	DQ218310	AY586709	USA	Larsson et al. (2004)
	*Resiniciumfriabile*	LWZ 20210923-23a	ON063692	ON427362	China	Wang et al. (2023)
-/Rickenellaceae	*Rickenelladanxiashanensis*	GDGM45513	MF326424	–	China	Zhang et al. (2018)
	*Rickenellafibula*	PBM 2503	DQ241782	MF318953	USA	Lutzoni et al. (2004)
-/Rigidoporaceae	*Leucophellinushobsonii*	Cui 6468	KT203288	KT203309	China	Direct Submission
	*Leucophellinusirpicoides*	Yuan 2690	KT203289	KT203310	China	Direct Submission
	*Rigidoporuscirratus*	LWZ 20170818-16	ON427472	ON427355	China	Wang et al. (2023)
	*Rigidoporuspopulinus*	LWZ 20190811-39a	ON063674	ON063874	China	Wang et al. (2023)
-/Schizocorticiaceae	*Schizocorticiumlenis*	LWZ 20180922-61	ON063698	ON063898	China	Wang et al. (2023)
	*Schizocorticiummagnosporum*	Wu 1510-34	MK405351	MK405337	China	Wu et al. (2021)
	*Schizocorticiummediosporum*	LWZ 20180921-7	ON063696	ON063896	China	Wang et al. (2023)
	*Schizocorticiummediosporum*	Chen 2456	MK405359	MK405345	China	Wu et al. (2021)
	*Schizocorticiumparvisporum*	GC 1508-127	MK405361	MK405347	China	Wu et al. (2021)
-/Schizoporaceae	*Fasciodontiabrasiliensis*	MSK-F 7245a	MK575201	MK598734	Brazil	Yurchenko et al. (2020)
-/Schizoporaceae	*Fasciodontiayunnanensis*	LWZ 20190811-50a	ON063675	ON427360	China	Wang et al. (2023)
	***Lyomycesalbofarinaceus***	CLZhao 33479*	**PQ523359**	–	**China**	**Present study**
	***Lyomycesalbofarinaceus***	**CLZhao 26661**	**PQ523360**	–	**China**	**Present study**
	*Lyomycesalbopulverulentus*	CLZhao 21478	OP730712	–	China	Guan et al (2023)
	*Lyomycesallantosporus*	KAS-GEL4933	KY800401	–	France	Yurchenko et al. (2017)
	*Lyomycesallantosporus*	FR-0249548	KY800397	–	France	Yurchenko et al. (2017)
	*Lyomycesaustro-occidentalis*	LWZ 20190816-40a	MZ262538	–	China	Liu et al. (2024)
	*Lyomycesbambusinus*	CLZhao 4831	MN945968	–	China	Chen and Zhao (2020)
	*Lyomycesbambusinus*	CLZhao 4808	MN945970	–	China	Chen and Zhao (2020)
	*Lyomycesboquetensis*	EYu 190727-12	PP471797	–	Panama	Yurchenko et al. (2024a)
	*Lyomycescremeus*	CLZhao 4138	MN945974	–	China	Chen and Zhao (2020)
	*Lyomycescremeus*	CLZhao 8295	MN945972	–	China	Chen and Zhao (2020)
	*Lyomycescrustosus*	TASM:YG G39	MF382993	–	Uzbekistan	Gafforov et al. (2017)
	*Lyomycescrustosus*	LWZ 20170815-23	MT319465	MT319201	China	Wang et al. (2021b)
	*Lyomycescrystallina*	LWZ 20190810-6b	OQ540901	–	China	Liu et al. (2024)
	*Lyomycesdaweishanensis*	CLZhao 18344	OR094474	–	China	Dong et al. (2024)
	*Lyomycesdensiusculus*	Ryvarden 44818	OK273853	–	Uganda	Viner et al. (2021)
	*Lyomycesdenudatus*	Ryvarden 19256	ON980759	–	Argentina	Viner and Miettinen (2022)
	*Lyomycesdenudatus*	Ryvarden 19436	ON980760	–	Argentina	Viner and Miettinen (2022)
	*Lyomyceselaeidicola*	LWZ20180411-20	MT319458	–	China	Wang et al. (2021b)
	*Lyomyceselaeidicola*	LWZ20180411-19	MT319457	–	China	Wang et al. (2021b)
	*Lyomyceserastii*	TASM:YG 022	MF382992	–	Uzbekistan	Gafforov et al. (2017)
	*Lyomyceserastii*	23cSAMHYP	JX857800	–	Spain	Unpublished
	*Lyomycesfimbriatus*	Wu910620-7	MK575209	–	China	Yurchenko et al. (2020)
	*Lyomycesfimbriatus*	Wu911204-4	MK575210	–	China	Yurchenko et al. (2020)
	***Lyomycesalbomarginatus***	CLZhao 22551*	**PQ644120**	**PQ644121**	**China**	**Present study**
	*Lyomycesfissuratus*	CLZhao 4352	MW713742	–	China	Luo et al. (2021b)
	*Lyomycesfissuratus*	CLZhao 4291	MW713738	–	China	Luo et al. (2021b)
	*Lyomycesfumosus*	CLZhao 8188	MW713744	–	China	Luo et al. (2021b)
	*Lyomycesgatesiae*	LWZ20180515-3	MT319447	–	China	Wang et al. (2021b)
	*Lyomycesgatesiae*	LWZ20180515-32	MT319448	–	China	Wang et al. (2021b)
	*Lyomycesgranulosus*	KAS-GEL1662	PP471799	–	Costa Rica	Yurchenko et al. (2024a)
	*Lyomycesgriseliniae*	KHL 12971 (GB)	DQ873651	–	Costa Rica	Larsson et al. (2006)
	*Lyomycesgriseliniae*	CBS:126042	MH864057	–	New Zealand	Vu et al. (2018)
	*Lyomycesguttulatus*	LWZ 20200921-29a	OQ540899	–	China	Liu et al. (2024)
	*Lyomycesguttulatus*	LWZ 20190810-20b	OQ540898	–	China	Liu et al. (2024)
-/Schizoporaceae	*Lyomyceshengduanensis*	CLZhao 20627	OR793233	–	China	Yuan and Zhao (2024)
	*Lyomyceshengduanensis*	CLZhao 25551	OR658999	–	China	Yuan and Zhao (2024)
	*Lyomycesincanus*	CLZhao 22813	OR094480	–	China	Dong et al. (2024)
	*Lyomycesincanus*	CLZhao 22900	OR094481	–	China	Dong et al. (2024)
	*Lyomycesjuniperi*	FR-0261086	KY081799	–	France	Riebesehl and Langer (2017)
	*Lyomycesleptocystidiatus*	LWZ 20170814-14	MT319429	MT319163	China	Wang et al. (2021b)
	*Lyomycesleptocystidiatus*	LWZ 20170818-1	MT326514	–	China	Wang et al. (2021b)
	*Lyomycesleptocystidiatus*	LWZ 20170818-2	MT326513	–	China	Wang et al. (2021b)
	*Lyomyceslincangensis*	CLZhao 22966	OR094487	–	China	Dong et al. (2024)
	*Lyomycesluteoalbus*	CLZhao 18211	OR094485	–	China	Dong et al. (2024)
	*Lyomycesluteoalbus*	CLZhao 18347	OR094486	–	China	Dong et al. (2024)
	*Lyomycesmacrosporus*	CLZhao 4516	MN945977	–	China	Chen and Zhao (2020)
	*Lyomycesmascarensis*	KAS-GEL4833	KY800399	–	France	Yurchenko et al. (2020)
	*Lyomycesmascarensis*	KAS-GEL4908	KY800400	–	France	Yurchenko et al. (2020)
	*Lyomycesmicrofasciculatus*	CLZhao 5109	MN954311	–	China	Chen and Zhao (2020)
	*Lyomycesnapoensis*	EYu 190720-18	PP471800	–	Ecuador	Yurchenko et al. (2024a)
	*Lyomycesneocrustosus*	EYu 190728-14	PP471801	–	Panama	Yurchenko et al. (2024a)
	*Lyomycesniveomarginatus*	CLZhao 16360	PP537949	–	China	Yuan and Zhao (2024)
	*Lyomycesniveus*	CLZhao 6431	MZ262541	–	China	Luo et al. (2021b)
	*Lyomycesniveus*	CLZhao 6442	MZ262542	–	China	Luo et al. (2021b)
	*Lyomycesochraceoalbus*	CLZhao 4385	MZ262535	–	China	Luo et al. (2021b)
	*Lyomycesochraceoalbus*	CLZhao 4725	MZ262536	–	China	Luo et al. (2021b)
	*Lyomycesoleifer*	KAS-Ec47	PP471802	–	Ecuador	Yurchenko et al. (2024a)
	*Lyomycesorarius*	EYu 190724-1	PP471805	–	Ecuador	Yurchenko et al. (2024a)
	*Lyomycesorganensis*	MSK7247	KY800403	–	Brazil	Yurchenko et al. (2017)
	*Lyomycesorientalis*	GEL3376	DQ340325	–	Germany	Yurchenko et al. (2017)
	*Lyomycespantropicus*	EYu 190727-23b	PP471808	–	Panama	Yurchenko et al. (2024a)
	*Lyomycesparvus*	KAS-GEL1599	PP471810	–	Costa Rica	Yurchenko et al. (2024a)
	*Lyomycespruni*	GEL2327	DQ340312	–	Germany	Larsson et al. (2006)
	*Lyomycespruni*	Ryberg 021018 (GB)	DQ873624	–	Sweden	Larsson et al. (2006)
	*Lyomycespunctatomarginatus*	CLZhao 22699	OR844492	–	China	Li et al. (2024b)
	*Lyomycespunctatomarginatus*	CLZhao 11629	OR844491	–	China	Li et al. (2024b)
	*Lyomycesqujingensis*	CLZhao 27462	OR167768	–	China	Dong et al. (2024)
	*Lyomycessambuci*	KAS-JR7	KY800402	–	Germany	Yurchenko et al. (2017)
-/Schizoporaceae	*Lyomycessambuci*	83SAMHYP	JX857721	–	Sweden	Yurchenko et al. (2017)
	*Lyomycessambuci*	LWZ 20180905-1	MT319444	MT319178	China	Wang et al. (2021b)
	*Lyomycessceptrifer*	KAS-Ec661	PP471811	–	Ecuador	Yurchenko et al. (2024a)
	*Lyomycessinensis*	CLZhao 27391	OR167769	–	China	Dong et al. (2024)
	*Lyomycessinensis*	CLZhao 27464	OR167770	–	China	Dong et al. (2024)
	*Lyomycessubcylindricus*	EYu 190727-25	PP471817	–	Panama	Yurchenko et al. (2024a)
	*Lyomycestasmanicus*	LWZ 20180515–17	OQ540900	–	China	Liu et al. (2024)
	*Lyomycesvietnamensis*	TNM F9073	JX175044	–	China	Yurchenko et al. (2017)
	*Lyomyceswuliangshanensis*	CLZhao 4108	MN945980	–	China	Chen and Zhao (2020)
	*Lyomyceswuliangshanensis*	CLZhao 4167	MN945979	–	China	Chen and Zhao (2020)
	*Lyomyceswumengshanensis*	CLZhao 29374	OR803021	–	China	Yuan and Zhao (2024)
	*Lyomyceswumengshanensis*	CLZhao 31486	OR899208	–	China	Yuan and Zhao (2024)
	*Lyomycesyunnanensis*	CLZhao 9375	OP730710	–	China	Guan et al. (2023)
	*Lyomycesyunnanensis*	CLZhao 10041	OP730709	–	China	Guan et al. (2023)
	*Lyomyceszhaotongensis*	CLZhao 32878	PP537950	–	China	Yuan et al. (2024)
	*Xylodonacuminatus*	Larsson 16029	ON197552	–	Brazil	Viner et al. (2023)
	*Xylodonacystidiatus*	LWZ 20180514-9	MT319474	–	China	Wang et al. (2021b)
	*Xylodonafromontanus*	O-F-904012	OQ645463	–	Rwanda	Yurchenko et al. (2024a)
	*Xylodonangustisporus*	Ryvarden 50691b	OK273831	–	Cameroon	Viner et al. (2021)
	*Xylodonapacheriensis*	Canfield 180	KY081800	–	USA	Wang et al. (2021b)
	*Xylodonasiaticus*	CLZhao 10368	OM959479	–	China	Unpublished
	*Xylodonasper*	Spirin 11923	OK273838	–	Russia	Viner et al. (2021)
	*Xylodonastrocystidiatus*	TNM F24764	NR154054	–	USA	Yurchenko and Wu (2014)
	*Xylodonattenuatus*	Spirin 8775	MH324476	–	Russia	Wang et al. (2021b)
	*Xylodonaustralis*	LWZ 20180509-8	MT319503	–	China	Wang et al. (2021b)
	*Xylodonbambusinus*	CLZhao 9174	MW394657	–	China	Ma and Zhao (2021)
	*Xylodonbamburesupinus*	CLZhao 23088	OR167773	–	China	Dong et al. (2024)
	*Xylodonborealis*	JS 26064	AY463429	–	Sweden	Larsson et al. (2004)
	*Xylodonbrevisetus*	JS 17863	AY463428	–	Sweden	Larsson et al. (2004)
	*Xylodoncremeoparinaceus*	CLZhao 23388	PP537951	–	China	Yuan and Zhao (2024)
	*Xylodoncrystalliger*	KUN 2312	NR166242	–	USA	Viner et al. (2018)
	*Xylodoncymosus*	Miettinen 19606	ON197554	–	Finland	Viner et al. (2023)
	*Xylodoncystidiatus*	FR-0249200	MH880195	–	Germany	Wang et al. (2021b)
	*Xylodondamansaraensis*	LWZ 20180417-23	MT319499	–	China	Wang et al. (2021b)
	*Xylodondaweishanensis*	CLZhao 18357	OP730715	–	China	Guan et al. (2023)
	*Xylodondetriticus*	Zíbarová 30.10.17	MH320793	–	Russia	Wang et al. (2021b)
	*Xylodondissiliens*	Ryvarden 44817	OK273856	–	Finland	Viner et al. (2021)
-/Schizoporaceae	*Xylodonechinatus*	OM 18237	OQ645464	–	Germany	Yurchenko et al. (2024a)
	*Xylodonfilicinus*	MSK-F 12869	MH880199	–	Germany	Wang et al. (2021b)
	*Xylodonfissilis*	CLZhao 18740	OR096211	–	China	Dong et al. (2024)
	*Xylodonfissuratus*	CLZhao 9407	OP730714	–	China	Guan et al. (2023)
	*Xylodonflaviporus*	FR-0249797	MH880201	–	Germany	Wang et al. (2021b)
	*Xylodonflocculosus*	CLZhao 18342	MW980776	–	China	Unpublished
	*Xylodonfollis*	FR-0249814	MH880204	–	Germany	Wang et al. (2021b)
	*Xylodongloeocystidiifer*	BLS M-5232	OQ645467	–	Germany	Yurchenko et al. (2024a)
	*Xylodongossypinus*	CLZhao 8375	MZ663804	–	China	Luo et al. (2021a)
	*Xylodongrandineus*	CLZhao 6425	OM338090	–	China	Luo et al. (2022)
	*Xylodonhastifer*	K(M) 172400	NR166558	–	USA	Riebesehl and Langer (2017)
	*Xylodonheterocystidiatus*	Wei 17-314	MT731753	–	China	Wu et al. (2021)
	*Xylodonhjortstamii*	Gorjon 3187	ON188816	–	Finland	Direct Submission
	*Xylodonhydnoides*	CLZhao 17991	OR096203	–	China	Dong et al. (2024)
	*Xylodonhyphodontinus*	KAS-GEL9222	MH880205	–	Germany	Riebesehl et al. (2019)
	*Xylodonjacobaeus*	MA-Fungi 91340	MH430073	–	Spain	Wang et al. (2021b)
	*Xylodonkunmingensis*	TUB-FO 42565	MH880198	–	Germany	Wang et al. (2021b)
	*Xylodonlaceratus*	CLZhao 9892	OL619258	–	China	Qu et al. (2022)
	*Xylodonlagenicystidiatus*	LWZ 20180515-14	MT319633	–	China	Wang et al. (2021b)
	*Xylodonlagenicystidiatus*	LWZ 20180513-16	MT319634	–	China	Wang et al. (2021b)
	*Xylodonlanatus*	CFMR FP-101864-A	OQ645474	–	Germany	Yurchenko et al. (2024a)
	*Xylodonlaxiusculus*	Ryvarden 44877	OK273827	–	Finland	Viner et al. (2021)
	*Xylodonlenis*	Wu 890714-3	KY081802	–	Germany	Yurchenko et al. (2024a)
	*Xylodonluteodontioides*	CLZhao 3207	MH114740	–	China	Yuan and Zhao (2024)
	*Xylodonluteodontioides*	CLZhao 18494	PP505422	–	China	Yuan and Zhao (2024)
	*Xylodonmacrosporus*	CLZhao 10226	MZ663809	–	China	Luo et al. (2021a)
	*Xylodonmagallanesii*	MA: Fungi:90397	MT158729	–	Spain	Fernandez-Lopez et al. (2020)
	*Xylodonmantiqueirensis*	MV 529	OQ645478	–	Germany	Yurchenko et al. (2024a)
	*Xylodonmollissimus*	LWZ 20160318-3	KY007517	–	China	Kan et al. (2017b)
	*Xylodonmontanus*	CLZhao 8179	OL619260	–	China	Qu et al. (2022)
	*Xylodonmuchuanensis*	LWZ 20200819-3a	OQ540903	–	China	Liu et al. (2024)
	***Xylodonmusicola***	CLZhao 35567*	**PQ523358**	–	**China**	**Present study**
	*Xylodonneotropicus*	MV 580	OQ645479	–	Germany	Yurchenko et al. (2024a)
	*Xylodonnesporii*	LWZ 20180921-35	MT319655	–	China	Wang et al. (2021b)
	*Xylodonnesporii*	LWZ 20190814-17a	ON063679	ON063879	China	Wang et al. (2023)
	*Xylodonniemelaei*	CLZhao 3746	MK269038	–	China	Unpublished
	*Xylodonnongravis*	GC 1412-22	KX857801	–	China	Chen et al. (2017)
	*Xylodonnothofagi*	ICMP 13842	AF145583	–	Sweden	Wang et al. (2021b)
	*Xylodonolivaceobubalinus*	CLZhao 25174	OR167772	–	China	Dong et al. (2024)
-/Schizoporaceae	*Xylodonovisporus*	LWZ 20170815-31	MT319666	–	China	Wang et al. (2021b)
	*Xylodonovisporus*	LWZ 20190817-6b	ON063680	ON063880	China	Wang et al. (2023)
	*Xylodonpapillosus*	CBS 114.71	MH860026	–	Germany	Vu et al. (2018)
	*Xylodonparadoxus*	Dai 14983	MT319519	–	China	Wang et al. (2021b)
	*Xylodonpatagonicus*	ICMP 13832	AF145581	–	Sweden	Wang et al. (2021b)
	*Xylodonpingbianensis*	CLZhao 19029	OR096208	–	China	Dong et al. (2024)
	*Xylodonporoides*	CLZhao 17845	PP505420	–	China	Yuan and Zhao (2024)
	*Xylodonpruinosus*	Spirin 2877	MH332700	–	Russia	Wang et al. (2021b)
	*Xylodonpruniaceus*	Ryvarden 11251	OK273828	–	Finland	Viner et al. (2021)
	*Xylodonpseudolanatus*	FP-150922	MH880220	–	Germany	Wang et al. (2021b)
	*Xylodonpseudotropicus*	Dai 10768	KF917543	–	China	Wang et al. (2021b)
	*Xylodonpuerensis*	CLZhao 8142	OP730720	–	China	Guan et al. (2023)
	*Xylodonpunctus*	CLZhao 17691	OM338092	–	China	Luo et al. (2022)
	*Xylodonpunctus*	CLZhao 17908	OM338093	–	China	Luo et al. (2022)
	*Xylodonpunctus*	CLZhao 17916	OM338094	–	China	Luo et al. (2022)
	*Xylodonquercinus*	Spirin 12030	OK273841	–	Finland	Viner et al. (2021)
	*Xylodonraduloides*	FCUG 2433	AF145570	–	Sweden	Wang et al. (2021b)
	*Xylodonramicida*	Spirin 7664	NR138013	–	Russia	Direct Submission
	*Xylodonreticulatus*	Wu 1109-178	KX857805	–	China	Wang et al. (2021b)
	*Xylodonreticulatus*	GC 1512-1	KX857808	–	China	Wang et al. (2021b)
	*Xylodonrimosissimus*	LWZ 20180904-28	ON063682	ON063882	China	Wang et al. (2023)
	*Xylodonrhizomorphus*	Dai 12367	NR154067	–	China	Zhao et al. (2014)
	*Xylodonrhododendricola*	LWZ 20180513-9	MT319621	–	China	Wang et al. (2021b)
	*Xylodonserpentiformis*	LWZ 20190816-12a	ON063683	ON063883	China	Wang et al. (2023)
	*Xylodonsinensis*	CLZhao 9197	MZ663810	–	China	Luo et al. (2021a)
	*Xylodonsinensis*	CLZhao 11120	MZ663811	–	China	Luo et al. (2021a)
	*Xylodonspathulatus*	LWZ 20180804-10	MT319646	–	China	Wang et al. (2021b)
	*Xylodonsubclavatus*	FO 42167	MH880232	–	China	Wang et al. (2021b)
	*Xylodonsubflaviporus*	TNM F29958	NR184880	–	USA	Chen et al. (2017)
	*Xylodonsubmucronatus*	Renvall 1602	OK273830	–	Finland	Viner et al. (2021)
	*Xylodonsubserpentiformis*	LWZ 20180512-16	MT319486	–	China	Wang et al. (2021b)
	*Xylodonsubtilissimus*	Spirin 12228	ON188818	–	Finland	Direct Submission
	*Xylodonsubtropicus*	LWZ 20180510-24	MT319541	–	China	Wang et al. (2021b)
	*Xylodontaiwanianus*	CBS 125875	MH864080	–	New Zealand	Vu et al. (2018)
	*Xylodontropicus*	CLZhao 3351	OL619261	–	China	Qu et al. (2022)
	*Xylodonussuriensis*	KUN 1989	NR166241	–	Russia	Direct Submission
	*Xylodonverecundus*	KHL 12261	DQ873642	–	USA	Wang et al. (2021b)
	*Xylodonvictoriensis*	LWZ 20180510-29	MT319487	–	China	Wang et al. (2021b)
	*Xylodonwenshanensis*	CLZhao 15729	OM338097	–	China	Luo et al. (2022)
	*Xylodonwumengshanensis*	CLZhao 32517	PP645439	–	China	Yuan and Zhao (2024)
	*Xylodonxinpingensis*	CLZhao 9174	MW394657	–	China	Ma and Zhao (2021)
	*Xylodonyarraensis*	LWZ 20180510-5	MT319639	–	China	Wang et al. (2021b)
	*Xylodonyunnanensis*	LWZ 20180922-47	MT319660	–	China	Wang et al. (2021b)
-/Sideraceae	*Sideralenis*	Miettinen 11036	FN907914	FN907914	Finland	Miettinen and Larsson (2011)
	*Sideraminutipora*	Cui 16720	MN621349	MN621348	Australia	Du et al. (2020b)
	*Siderasrilankensis*	Dai 19654	MN621344	MN621346	Sri Lanka	Du et al. (2020b)
	*Sideratenuis*	Dai 18697	MK331865	MK331867	Singapore	Liu et al. (2022)
	*Sideratibetica*	Dai 21057	MW198484	MW192009	Belarus	Liu et al. (2022)
-/Skvortzoviaceae	*Skvortzoviadabieshanensis*	LWZ 20210918-15b	ON063694	ON063894	China	Wang et al. (2023)
	*Skvortzoviapinicola*	LWZ 20210623-18b	ON063695	ON063895	China	Wang et al. (2023)
	*Skvortzoviaqilianensis*	LWZ 20180904-20	ON063693	ON063893	China	Wang et al. (2023)
	*Skvortzoviayunnanensis*	CLZhao 16084	MW472754	MW473473	China	Dong et al. (2021)
-/Tubulicrinaceae	*Tubulicrinisaccedens*	ACD0414	OL756001	–	USA	Unpublished
	***Tubulicrinisalbobadius***	CLZhao 26202*	**PQ523361**	**PQ523364**	**China**	**Present study**
	***Tubulicrinisalbobadius***	**CLZhao 26330**	**PQ523362**	**PQ523365**	**China**	**Present study**
	*Tubulicrinisaustralis*	MA Fungi:88838	KX017591	–	Spain	Unpublished
	*Tubulicrinisaustralis*	MA Fungi:88839	KX017593	–	Chile	Unpublished
	*Tubulicrinisborealis*	DK14_93	OL436811	–	USA	Unpublished
	*Tubulicriniscalothrix*	LWZ 20210919-1b	ON063704	–	China	Wang et al. (2023)
	*Tubulicrinischaetophorus*	Spirin 12616 (H)	ON188814	–	Slovenia	Direct Submission
	*Tubulicrinischaetophorus*	UC2023055	KP814255	–	USA	Rosenthal et al. (2017)
	*Tubulicrinischaetophorus*	UC2023059	KP814233	–	USA	Rosenthal et al. (2017)
	*Tubulicrinisglebulosus*	LWZ 20180903-13	ON063705	ON063905	China	Wang et al. (2023)
	*Tubulicrinisglebulosus*	DK16_14	OL436905	–	USA	Unpublished
	*Tubulicrinisglebulosus*	UC2023229	KP814463	–	Canada	Rosenthal et al. (2017)
	*Tubulicrinisglobisporus*	KHL 12133 (GB)	DQ873655	–	Sweden	Larsson et al. (2006)
	*Tubulicrinisgracillimus*	PDD 95851	HQ533047	–	New Zealand	Unpublished
	*Tubulicrinishirtellus*	KHL 11717 (GB)	DQ873657	–	Sweden	Larsson et al. (2006)
	*Tubulicrinisinornatus*	KHL 11763 (GB)	DQ873659	–	Sweden	Larsson et al. (2006)
	*Tubulicrinisinornatus*	OTU782	MT596347	–	Japan	Unpublished
	*Tubulicrinismartinicensis*	GG-MAR12-206	NR_163282	–	France	Unpublished
	*Tubulicrinispini*	CLZhao 6881	OR096210	–	China	Dong et al. (2024)
	*Tubulicrinissubulatus*	UC2023072	KP814430	–	USA	Rosenthal et al. (2017)
	*Tubulicrinissubulatus*	LWZ 20190914-7	ON063706	ON063906	China	Wang et al. (2023)
	*Tubulicrinisxantha*	CLZhao 2868	MT153874	–	China	He et al. (2020)
	*Tubulicrinisxantha*	CLZhao 2869	MT153875	–	China	He et al. (2020)
	*Tubulicrinisyunnanensis*	CLZhao 3418	MT153879	–	China	He et al. (2020)
	*Tubulicrinisyunnanensis*	CLZhao 9717	MT153880	–	China	He et al. (2020)
-/Umbellaceae	*Umbellussinensis*	LWZ 20190615-27	OR242616	OR236212	China	Wang and Zhou (2024)
	*Umbellussinensis*	LWZ 20190615-39	OR242617	OR236213	China	Wang and Zhou (2024)
-/Incertae sedis	*Alloclavariapurpurea*	M. Korhonen 10305	MF319044	MF318895	Finland	Unpublished
	*Athelodermamirabile*	TAA 169235	DQ873592	DQ873592	Estonia	Larsson et al. (2006)
-/Incertae sedis	*Blasiphaliapseudogrisella*	P. Joijer 4118	MF319047	MF318898	Finland	Unpublished
	*Bryopistillariasagittiformis*	IO.14.164	MT232349	MT232303	Sweden	Olariaga et al. (2020)
	*Cantharellopsisprescotii*	H6059300	MF319051	MF318903	Finland	Unpublished
	*Contumycesvesuvianus*	203608	–	MF318913	Italy	Unpublished
	*Ginnsiaviticola*	Wu 0010-29	MN123802	GQ470670	China	Wu et al. (2021)
	*Globuliciumhiemale*	Hjm 19007	DQ873595	DQ873595	Sweden	Larsson et al. (2006)
	*Gyroflexusbrevibasidiata*	IO.14.230	MT232351	MT232305	Sweden	Olariaga et al. (2020)
	*Hastodontiahalonata*	HHB-17058	MK575207	MK598738	Mexico	Yurchenko et al. (2020)
	*Hastodontiahastata*	KHL 14646	MH638232	MH638232	Norway	Larsson (2007)
	*Lawrynomycescapitatus*	KHL 8464	DQ677491	DQ677491	Sweden	Larsson (2007)
	*Loreleiamarchantiae*	Lutzoni 930826-1	U66432	U66432	USA	Lutzoni F (1997)
	*Lyoathelialaxa*	Spirin 8810a	MT305998	MT305998	USA	Sulistyo et al. (2021)
	*Muscinuptalaevis*	V. Haikonen 19745	MF319066	MF318921	Finland	Unpublished
	*Sphaerobasidiumminutum*	KHL 11714	DQ873652	DQ873653	Finland	Larsson et al. (2006)
	*Tsugacorticiumkenaicum*	CFMR HHB17347	–	JN368221	USA	Nakasone (2012)
Polyporales/ Fomitopsidaceae	*Fomitopsispinicola*	AFTOL 770	AY854083	AY684164	USA	Lutzoni et al. (2004)
-/Grifolaceae	*Grifolafrondosa*	AFTOL 701	AY854084	AY629318	USA	Lutzoni et al. (2004)
-/Thelephoraceae	*Thelephoraganbajun*	ZRL20151295	LT716082	KY418908	China	Zhao et al. (2017)

Maximum parsimony (MP), Maximum Likelihood (ML), and Bayesian Inference (BI) analyses were applied to the combined three datasets following a previous study (Zhao and Wu 2017), and the tree construction procedure was performed in PAUP* version 4.0b10 (Swofford 2002). All of the characters were equally weighted, and gaps were treated as missing data. Using the heuristic search option with TBR branch swapping and 1,000 random sequence additions, trees were inferred. Max trees were set to 5,000, branches of zero length were collapsed, and all parsimonious trees were saved. Clade robustness was assessed using bootstrap (BT) analysis with 1,000 replicates (Felsenstein 1985). Descriptive tree statistics, tree length (TL), consistency index (CI), retention index (RI), rescaled consistency index (RC), and homoplasy index (HI) were calculated for each maximum parsimonious tree generated. The multiple sequence alignments were also analyzed using maximum likelihood (ML) in RAxML-HPC2 on XSEDE v 8.2.8 with default parameters (Miller et al 2012). Branch support (BS) for ML analysis was determined by 1,000 bootstrap replicates.

jModelTest v2 (Darriba et al. 2012) was used to determine the best-fit evolution model for each dataset for the purposes of Bayesian inference (BI), Bayesian inference was performed using MrBayes 3.2.7a with a GTR+I+G model of DNA substitution and a gamma distribution rate variation across sites (Ronquist et al. 2012). The first one-fourth of all the generations were discarded as burn-ins. The majority-rule consensus tree of all the remaining trees was calculated. Branches were considered significantly supported if they received a maximum likelihood bootstrap value (BS) of ≥ 70%, a maximum parsimony bootstrap value (BT) of ≥ 70%, or Bayesian posterior probabilities (BPP) of ≥ 0.95.

## ﻿Results

### ﻿Sequence similarity search

The ITS+nLSU dataset (Fig. 1) comprised 80 specimens representing 77 species of the phylogeny of the order Hymenochaetales. The dataset had an aligned length of 2,548 characters, of which 1,018 characters are constant, 476 are variable and parsimony uninformative, and 1,054 are parsimony informative. Maximum parsimony analysis yielded 1 equally parsimonious tree (TL = 10768, CI = 0.2612, HI = 0.7388, RI = 0.4087, and RC = 0.1068). The best model for the ITS+nLSU dataset, estimated and applied in the Bayesian analysis, was GTR+I+G. Both Bayesian analysis and ML analysis resulted in a similar topology to MP analysis with an average standard deviation of split frequencies = 0.044070 (BI), and the effective sample size (ESS) for Bayesian analysis across the two runs is double the average ESS (avg ESS) = 645.

The ITS dataset (Fig. 2) comprised 78 specimens representing 69 species of the one new species and related species in the genus *Hymenochaete*. The dataset had an aligned length of 470 characters, of which 209 characters are constant, 35 are variable and parsimony uninformative, and 226 are parsimony informative. Maximum parsimony analysis yielded 68 equally parsimonious trees (TL = 1574, CI = 0.3018, HI = 0.6982, RI = 0.6876, and RC = 0.2075). The best model for the ITS dataset, estimated and applied in the Bayesian analysis, was GTR+I+G. Both Bayesian analysis and ML analysis resulted in a similar topology to MP analysis with an average standard deviation of split frequencies = 0.012875 (BI), and the effective sample size (ESS) for Bayesian analysis across the two runs is double the average ESS (avg ESS) = 269.5.

The ITS dataset (Fig. 3) comprised 81 specimens representing 56 species of two new species and related taxa in the genus *Lyomyces*. The dataset had an aligned length of 470 characters, of which 209 characters are constant, 35 are variable and parsimony uninformative, and 226 are parsimony informative. Maximum parsimony analysis yielded 6 equally parsimonious trees (TL = 1574, CI = 0.3018, HI = 0.6982, RI = 0.6876, and RC = 0.2075). The best model for the ITS dataset, estimated and applied in the Bayesian analysis, was GTR+I+G. Both Bayesian analysis and ML analysis resulted in a similar topology to MP analysis with an average standard deviation of split frequencies = 0.012875 (BI), and the effective sample size (ESS) for Bayesian analysis across the two runs is double the average ESS (avg ESS) = 269.5.

The ITS dataset (Fig. 4) comprised 28 specimens representing 17 species of new species and related taxa in the genus *Tubulicrinis*. The dataset had an aligned length of 772 characters, of which 284 characters are constant, 152 are variable and parsimony uninformative, and 336 are parsimony informative. Maximum parsimony analysis yielded 2 equally parsimonious trees (TL = 1409, CI = 0.5777, HI = 0.4223, RI = 0.6248, and RC = 0.3610). The best model for the ITS dataset, estimated and applied in the Bayesian analysis, was GTR+I+G. Both Bayesian analysis and ML analysis resulted in a similar topology to MP analysis with an average standard deviation of split frequencies = 0.006683 (BI), and the effective sample size (ESS) for Bayesian analysis across the two runs is double the average ESS (avg ESS) = 633.

The ITS dataset (Fig. 5) comprised 104 specimens representing 97 species of the new species and related taxa in the genus *Xylodon*. The dataset had an aligned length of 673 characters, of which 233 characters are constant, 81 are variable and parsimony uninformative, and 359 are parsimony informative. Maximum parsimony analysis yielded 5,000 equally parsimonious trees (TL = 3842, CI = 0.2140, HI = 0.7860, RI = 0.4337, and RC = 0.0928). The best model for the ITS dataset, estimated and applied in the Bayesian analysis, was GTR+I+G. Both Bayesian analysis and ML analysis resulted in a similar topology to MP analysis with an average standard deviation of split frequencies = 0.022556 (BI), and the effective sample size (ESS) for Bayesian analysis across the two runs is double of the average ESS (avg ESS) = 1143.5.

The phylogram based on the combined ITS+nLSU sequences (Fig. 1) analysis showed that five new species *Hymenochaeteweishanensis*, *Lyomycesalbofarinaceus*, *Lyomycesalbomarginatus*, *Tubulicrinisalbobadius* and *Xylodonmusicola* were assigned to the genera *Hymenochaete*, *Lyomyces*, *Tubulicrinis* and *Xylodon* within the order Hymenochaetales, individually. The phylogenetic tree based on ITS sequences (Fig. 2), revealed that *H.weishanensis* was retrieved as a sister to *H.luteobadia*. The taxon based on the ITS sequences (Fig. 3) revealed that *L.albofarinaceus* was grouped with *L.albopulverulentus* and *L.qujingensis*. *L.albomarginatus* was sister to *L.crustosus*. The topology based on the ITS sequences (Fig. 4), revealed that *T.albobadius* was grouped with *T.australis* and *T.inornatus*. The phylogenetic tree, based on ITS sequences (Fig. 5), revealed that *X.musicola* grouped with three taxa: *X.gloeocystidiifer*, *X.hydnoides*, and *X.neotropicus*.

### ﻿Taxonomy

#### ﻿ Hymenochaete weishanensis

Taxon classificationFungiHymenochaetalesHymenochaetaceae

Y.F. Dai & C.L. Zhao sp. nov.

56049EB6-8B30-547E-868E-FFBC99D86640

856316

Figs 6 , 7

##### Holotype.

China • Yunnan Province, Dali, Weishan County, Leqiu Town, Zhongyao Village, GPS coordinates 25°02′N, 100°16′E, evel. 1910 m a.s.l., on a fallen branch of angiosperm, leg. C.L. Zhao, 19 July 2022, CLZhao 22615 (SWFC).

**Figure 6.**
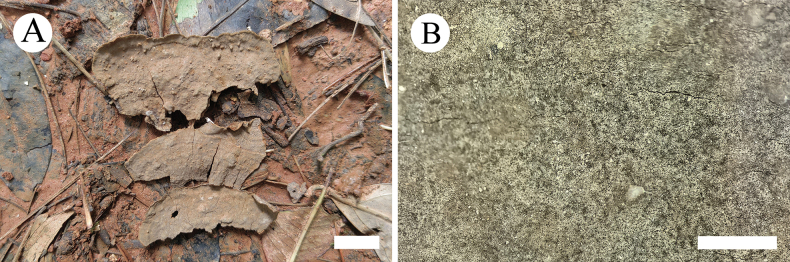
Basidiomata of *Hymenochaeteweishanensis* in general and detailed views (CLZhao 22615, holotype). Scale bars: 1cm (**A**); 1mm (**B**).

##### Etymology.

*weishanensis* (Lat.), refers to the locality (Weishan) of the holotype.

**Figure 7.**
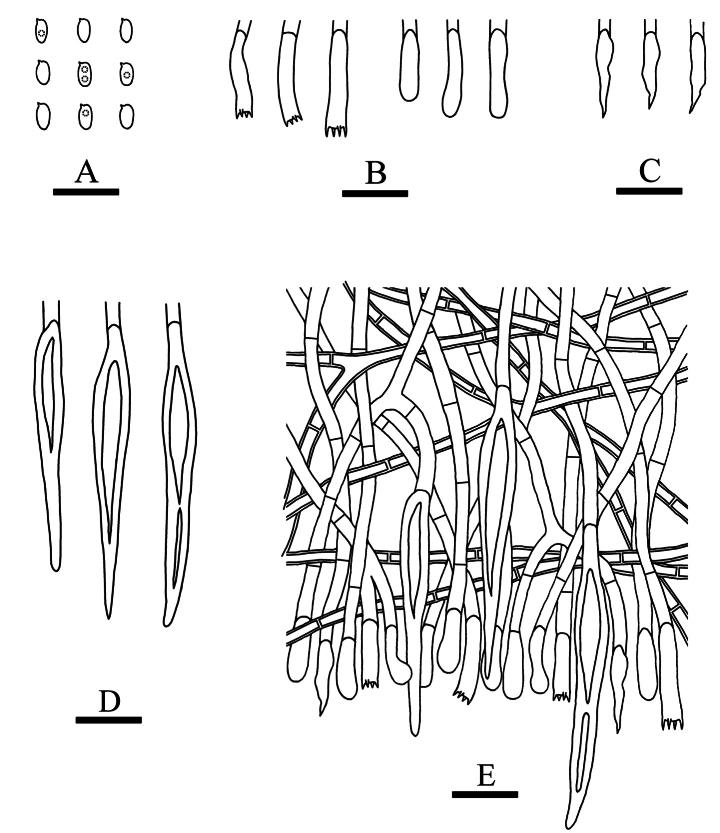
Microscopic structures of *Hymenochaeteweishanensis* (holotype, CLZhao 22615). **A** Basidiospores **B** basidia and basidioles **C** tapering cystidia **D** setae **E** part of the vertical section of hymenium. Scale bars: 10 µm (**A–E**).

##### Basidiomata.

Annual, effused-reflexed, thin, coriaceous, without odor or taste when fresh, up to 5 cm long, 2.5 cm wide, and 150 µm thick. Pileal surface dark brown upon drying. Hymenial surface tuberculate, lightly brown when fresh, turning to gray brown upon drying. Sterile margin narrow, slightly gray-brown, up to 1 mm wide.

##### Hyphal system.

Monomitic, generative hyphae with simple-septa, colorless, thin to slightly thick-walled, frequently branched, interwoven, 2.0–2.8 µm in diameter, IKI–, CB–; tissues unchanged in KOH.

##### Hymenium.

Cystidia tapering, thin-walled, smooth, 9.5–20.5 × 2.0–3.5 µm; cystidioles absent. Hymenial setae abundant, subulate, reddish brown, thick-walled, smooth, 33.0–61.5 × 5.0–8.5 µm, projecting above the hymenium. Basidia subclavate, colorless, thin-walled, simple-septum, with four sterigmata, 6.5–24.0 × 2.5–4.0 µm; basidioles in shape similar to basidia, but slightly smaller.

##### Basidiospores.

Elipsoid to narrow ellipsoid, colorless, thin-walled, smooth, with one or two guttate, IKI–, CB–, 4.0–5.0(–5.5) × 2.0–3.0 µm, L = 4.76 µm, W = 2.52 µm, Q = 1.86–1.92 (n = 60/2).

##### Additional specimens examined (Paratype).

China • Yunnan Province, Dali, Weishan County, Leqiu Town, Zhongyao Village, on a fallen angiosperm branch, 19 July 2022, CLZhao 40297 (SWFC).

#### ﻿ Lyomyces albofarinaceus

Taxon classificationFungiHymenochaetalesCorticiaceae

Y.F. Dai & C.L. Zhao sp. nov.

7C55DC5F-6E5A-5556-A9A5-7816D095F09D

856317

Figs 8 , 9

##### Holotype.

China • Yunnan Province, Zhaotong, Wumengshan National Nature Reserve, GPS coordinates 28°05′N, 104°20′E, evel. 1600 m a.s.l., on a fallen branch of angiosperm, leg. C.L. Zhao, 20 September 2023, CLZhao 33479 (SWFC).

**Figure 8.**
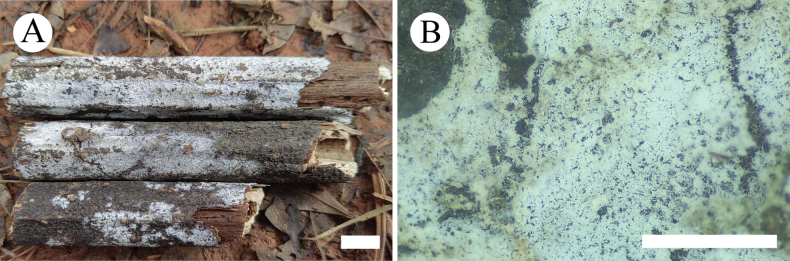
Basidiomata of *Lyomycesalbofarinaceus* in general and detailed views (CLZhao 33479, holotype). Scale bars: 1cm (**A**); 1mm (**B**).

##### Etymology.

*albofarinaceus* (Lat.), refers to the white and pruinose hymenophore surface.

**Figure 9.**
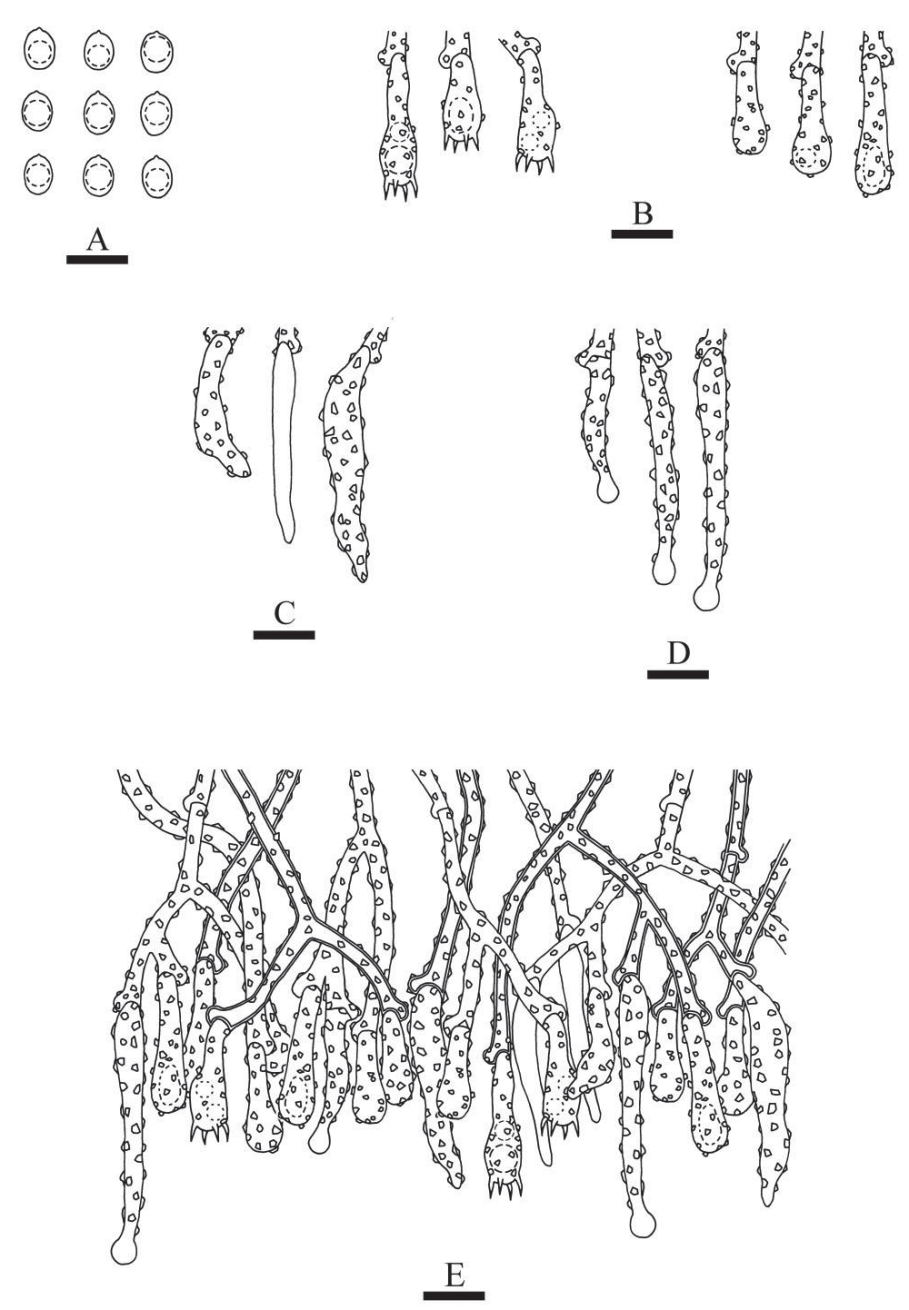
Microscopic structures of *Lyomycesalbofarinaceus* (holotype, CLZhao 33479). **A** Basidiospores **B** basidia and basidioles **C** tapering cystidia **D** Capitate cystidia **E** part of the vertical section of hymenium. Scale bars: 10 µm (**A–E**).

##### Basidiomata.

Annual, resupinate, adnate, without odor or taste when fresh, up to 8 cm long, 3.5 cm wide, and 150 µm thick. Hymenial surface pruinose, smooth, white when fresh, to white to cream upon drying. Sterile margin narrow, white, up to 1 mm wide.

##### Hyphal system.

Monomitic, generative hyphae with clamp connections, colorless, thin to slightly thick-walled, frequently branched, interwoven, 2.0–3.0 µm in diameter, IKI–, CB–; tissues unchanged in KOH, subhymenial hyphae densely covered by crystals.

##### Hymenium.

Cystidia of two types: (1) tapering, thin-walled, smooth to be covered by crystals, 11.5–44.0 × 4.5–7.5 µm; (2) capitate, thin-walled, smooth to be covered by crystals, slightly constricted at the neck, with a globose tip, 23.5–40.0 × 3.5–5.5 µm; cystidioles absent. Basidia clavate, colorless, thin-walled, with four sterigmata, 15.0–27.0 × 5.0–9.5 µm; basidioles in shape similar to basidia, but slightly smaller.

##### Basidiospores.

Broadly ellipsoid, colorless, thin-walled, smooth, with one guttate, IKI–, CB–, (5.5–)6.0–7.0(–7.5) × (4.5–)5.0–6.0(–6.5) µm, L = 6.46 µm, W = 5.62 µm, Q = 1.07–1.15 (n = 60/2).

##### Additional specimens examined (Paratype).

China • Yunnan Province, Qujing, Qilin District, Cuishan Forestry Park, on a fallen angiosperm branch, 5 November 2022, CLZhao 26661 (SWFC).

#### ﻿ Lyomyces albomarginatus

Taxon classificationFungiHymenochaetalesCorticiaceae

Y.F. Dai & C.L. Zhao sp. nov.

812ED088-29F1-5979-84A7-EACC79ACBDB3

856737

Figs 10 , 11

##### Holotype.

China • Yunnan Province, Dali, Weishan County, QinghuaTown, Green Peacock Reserve, Jiangzui Village, GPS coordinates 25°01′N, 100°11′E, evel. 1500 m a.s.l., on a fallen branch of angiosperm, leg. C.L. Zhao, 18 July 2022, CLZhao 22551 (SWFC).

**Figure 10.**
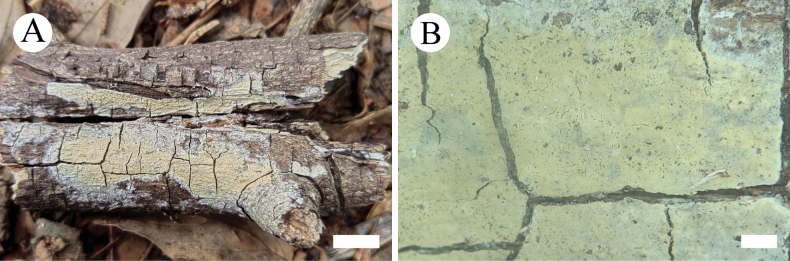
Basidiomata of *Lyomycesalbomarginatus* in general and detailed views (CLZhao 22551, holotype). Scale bars: 1cm (**A**); 1mm (**B**).

##### Etymology.

*albomarginatus* (Lat.), refers to the white margin of the basidiomata.

**Figure 11.**
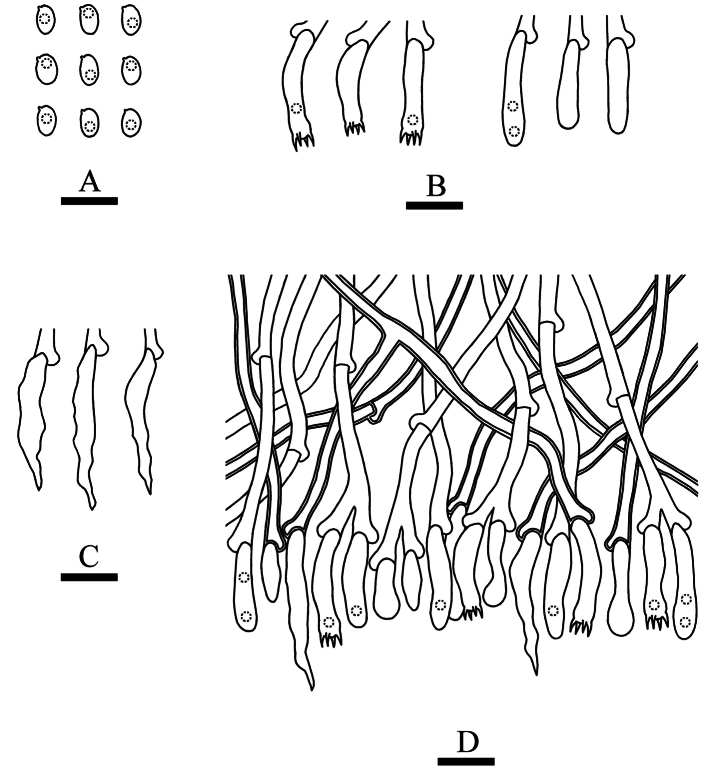
Microscopic structures of *Lyomycesalbomarginatus* (holotype, CLZhao 22551). **A** Basidiospores **B** basidia and basidioles **C** tapering cystidia **D** part of the vertical section of hymenium. Scale bars: 10 µm (**A–D**).

##### Basidiomata.

Annual, resupinate, adnate, without odor or taste when fresh, up to 8 cm long, 3 cm wide, and 150 µm thick. Hymenial surface cracked, slightly buff when fresh, turning to buff to slightly yellowish upon drying. Sterile margin slightly buff, up to 3 mm wide.

##### Hyphal system.

Monomitic, generative hyphae with clamp connections, colorless, thin to slightly thick-walled, rarely branched, interwoven, 2.0–3.5 µm in diameter, IKI–, CB–; tissues unchanged in KOH.

##### Hymenium.

Cystidia numerous, tapering, thin-walled, smooth, 22.5–30.0 × 2.0–4.0 µm; cystidioles absent. Basidia cylindrical, colorless, thin-walled, with four sterigmata, 15.0–19.0 × 3.5–4.3 µm; basidioles in shape similar to basidia, but slightly smaller.

##### Basidiospores.

Elliposoid, colorless, thin-walled, smooth, with one guttate, IKI–, CB–, (3.5–)4.0–5.5(–6.0) × (2.5–)2.7–3.5(–3.7) µm, L = 4.89 µm, W = 3.13 µm, Q = 1.56 (n = 30/1).

#### ﻿ Tubulicrinis albobadius

Taxon classificationFungiHymenochaetalesHymenochaetaceae

Y.F. Dai & C.L. Zhao sp. nov.

5568C8FB-0A4F-5881-B901-A71DBF47CCB1

856318

Figs 12 , 13

##### Holotype.

China • Yunnan Province, Qujing, Qilin District, Cuishan Forest Park, GPS coordinates 25°32′N, 103°42′E, evel. 2250 m a.s.l., on a fallen branch of angiosperm, leg. C.L. Zhao, 5 Novermber 2022, CLZhao 26202 (SWFC).

**Figure 12.**
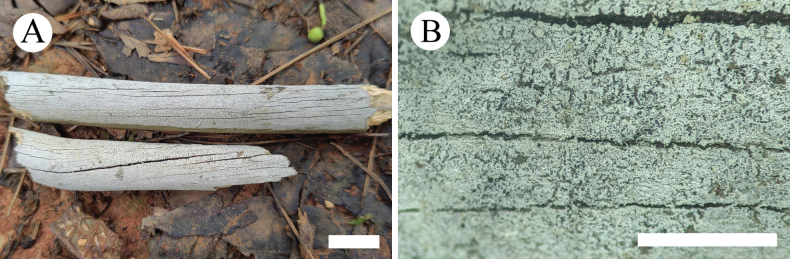
Basidiomata of *Tubulicrinisalbobadius* in general and detailed views (CLZhao 26202, holotype). Scale bars: 1cm (**A**); 1mm (**B**).

##### Etymology.

*albobadius* (Lat.), refers to the white basidiomata.

**Figure 13.**
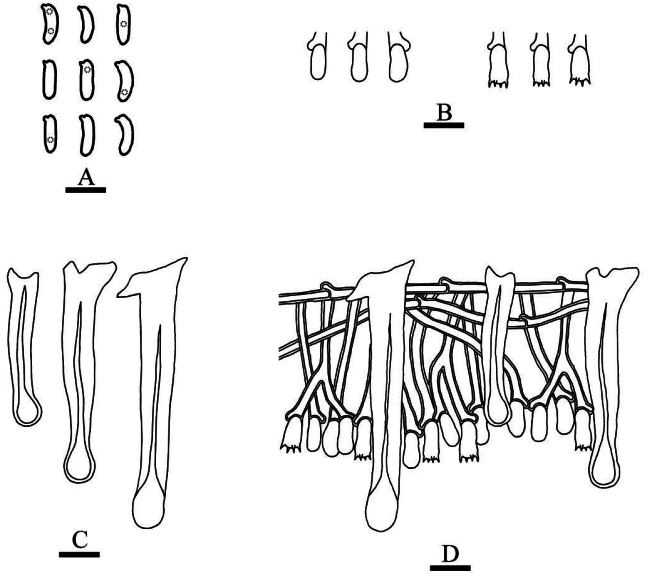
Microscopic structures of *Tubulicrinisalbobadius* (holotype, CLZhao 26202). **A** Basidiospores **B** basidia and basidioles **C** lyocystidia **D** part of the vertical section of hymenium. Scale bars: 5 µm (**A**); 10 µm (**B–D**).

##### Basidiomata.

Annual, resupinate, thin, adnate, without odor or taste when fresh, up to 8 cm long, 1.5 cm wide, and 150 µm thick. Hymenial surface arachnoid, white when fresh and became white to gray when drying. Sterile margin narrow, white, up to 1 mm.

##### Hyphal system.

Monomitic, generative hyphae with clamp connections, colorless, thick-walled, branched, interwoven, 2.0–4.0 µm in diameter, IKI–, CB–; tissues unchanged in KOH.

##### Hymenium.

Cystidia and cystidioles absent. Lyocystidia projecting, thick-walled, with a globose tip, some of the globose tips are thin-walled, 38.0–71.0 × 8.3–10.0 µm. Basidia barred, colorless, thin-walled, with four sterigmata, 9.5–14.0 × 4.0–5.0 µm; basidioles in shape similar to basidia, but slightly smaller.

##### Basidiospores.

Cylindrical to allantoid, colorless, thin-walled, smooth, with one or two guttate, IKI–, CB–, (3.5–)4.0–6.0(–6.5) × 1.5–2.2(–2.5) µm, L = 5.09 µm, W = 1.89 µm, Q = 2.55–2.77 (n = 60/2).

##### Additional specimens examined (Paratype).

China • Qujing, Qilin District, Cuishan Forest Park, on a fallen angiosperm branch, 5 November 2022, CLZhao 26330 (SWFC).

#### ﻿ Xylodon musicola

Taxon classificationFungiHymenochaetalesSchizoporaceae

Y.F. Dai & C.L. Zhao sp. nov.

43285015-F6A5-587F-A71A-B5714011A73D

856319

Figs 14 , 15

##### Holotype.

China • Yunnan Province, Zhaotong, Yongshan County, Mugan Town, Wumengshan Nature Reserve, GPS coordinates 28°05′N, 103°58′E, evel. 2200 m a.s.l., on a fallen angiosperm branch, leg. C.L. Zhao, 7 November 2023, CLZhao 35567 (SWFC).

**Figure 14.**
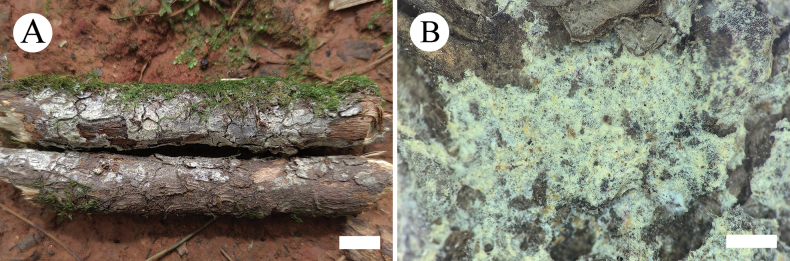
Basidiomata of *Xylodonmusicola* in general and detailed views (CLZhao 35567, holotype). Scale bars: 1cm (**A**); 1mm (**B**).

##### Etymology.

*musicola* (Lat.), refers to the growth on the mosses, which is located Bryophyta.

**Figure 15.**
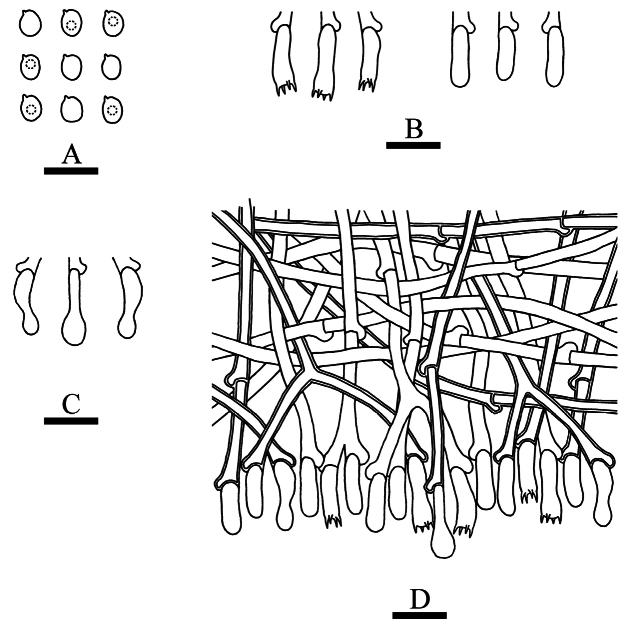
Microscopic structures of *Xylodonmusicola* (holotype, CLZhao 35567). **A** Basidiospores **B** basidia and basidioles **C** capitate cystidia **D** part of the vertical section of hymenium. Scale bars: 10 µm (**A–D**).

##### Basidiomata.

Annual, resupinate, adnate, very difficult to separate from substrate, without odor or taste when fresh, up to 7 cm long, 2 cm wide, and 150 µm thick. Hymenial surface arachnoid, white when fresh and becoming white to cream when drying. Sterile margin narrow, slightly cream, up to 1 mm wide. The basidiomata grow on the surface of muscus.

##### Hyphal system.

Monomitic, generative hyphae with clamp connections, colorless, thin to slightly thick-walled, rarely branched, interwoven, 2.5–4.0 µm in diam, IKI–, CB–; tissues unchanged in KOH.

##### Hymenium.

Cystidia capitate, thin-walled, smooth, slightly constricted at the neck, with a globose tip, 12.5–20.0 × 3.5–5.0 µm; cystidioles absent. Basidia clavate, colorless, thin-walled, with four sterigmata, 11.0–15.5 × 3.5–5.0 µm; basidioles in shape similar to basidia, but slightly smaller.

##### Basidiospores.

Broadly ellipsoid to globe, colorless, thin-walled, smooth, with one guttate, IKI–, CB–, 4.0–5.5(–6.0) × 3.5–5.0(–5.5) µm, L = 4.77 µm, W = 4.35 µm, Q = 1.07–1.13 (n = 60/2).

##### Additional specimens examined (Paratype).

China • Yunnan Province, Zhaotong, Yongshan County, Mugan Town, Wumengshan Nature Reserve, on a fallen angiosperm branch, 7 November 2023, CLZhao 40298 (SWFC).

## ﻿Discussion

The order Hymenochaetales comprises many representative corticioid fungal taxa, including hydnoid, corticioid, and polyporoid fungi possessing basidiomata with diverse hymenophoral and cystidial morphology (Riebesehl et al. 2019; Wu et al. 2022; Guan et al. 2023; Zhang et al. 2024). In the present study, five new species *Hymenochaeteweishanensis*, *Lyomycesalbofarinaceus*, *L.albomarginatus*, *Tubulicrinisalbobadius*, and *Xylodonmusicola* are described based on the phylogenetic analyses and morphological characteristics.

Based on ITS topology (Fig. 2), *Hymenochaeteweishanensis* grouped closely with two species *H.luteobadia* and *H.anomala*. However, *H.anomala* can be delimited from *H.weishanensis* by its smooth hymenial surface and narrower basidiospores (3.5–4.5 × 1.8–2.3 µm; Parmasto 2001). *Hymenochaeteluteobadia* differs from *H.weishanensis* due to its sulcate and zonate hymenial surface and wider basidia (15.0–20.0 × 4.0–5.0 µm; Parmasto 2001). Based on ITS topology (Fig. 3), the taxon *Lyomycesalbofarinaceus* grouped closely with *L.albopulverulentus* and *L.qujingensis*. The taxon *L.albomarginatus* was sister to *L.crustosus*. However, *L.qujingensis* differs from *L.albofarinaceus* due to its longer capitate cystidia (40.0–60.0 × 4.0–7.5 µm) and by possessing clavate cystidia (16.5–18.0 × 7.5–8.5 µm; Dong et al. 2024). *L.albopulverulentus* can be delimited from *L.albofarinaceus* by its longer basidiospores (8.0–10.5 × 5.5–7.0 µm; Guan et al. 2023). *Lyomycescrustosus* can be delimited from *L.albomarginatus* due to its odontoid hymenial surface and longer basidia (20–30 × 4.0–5.0 µm; Maekawa 1994). Based on ITS topology (Fig. 4), the taxon *Tubulicrinisalbobadius* grouped closely with *T.australis* and *T.inornatus*. However, *T.inornatus* differs from *T.albobadius* by its reticulate to porulose hymenial surface and wider basidiospores (4.0–5.0 × 2.5–3.5 µm; Maekawa 2021). Based on ITS topology (Fig. 5), the taxon *Xylodonmusicola* grouped closely with *X.gloeocystidiifer*, *X.hydnoides* and *X.neotropicus*. However, morphologically, *X.gloeocystidiifer* differs from *X.musicola* by its odontioid hymenial surface and smaller basidiospores (3.5–4.0 × 2.8–3.5 µm; Yurchenko et al. 2024b), *X.hydnoides* differs from *X.musicola* by its hydnoid hymenial surface and by possessing clavate cystidia (29.5–38.5 × 3.5–4.5 µm; Dong et al. 2024). *Xylodonneotropicus* can be delimited from *X.musicola* due to its odontioid to short hydnoid hymenial surface and shorter basidiospores (3.5–4.0 × 3.3–3.7 µm; Yurchenko et al. 2024b).

Morphologically, *Hymenochaeteweishanensis* resembles *H.colliculosa* (Sacc.) Parmasto, *H.biformisetosa* Jiao Yang & S.H. He and *H.sharmae* Hembrom, K. Das & A. Parihar by ellipsoid basidiospores. However, *H.colliculosa* differs from *H.weishanensis* due to its dimitic hyphal system and wider basidia (20.0–24.0 × 5.0–6.0 µm; He et al. 2017). The species *H.biformisetosa* differs from *H.weishanensis* by its smooth hymenial surface and wider basidiospores (4.3–6.0 × 3.0–4.2 µm; Yang and He 2014). The taxon *H.sharmae* differs from *H.weishanensis* by its smooth hymenial surface and wider basidia (12.0–16.0 × 4.0–6.0 µm; Wang et al. 2019)

Morphologically, *Lyomycesalbofarinaceus* resembles *L.incanus* J.H. Dong & C.L. Zhao, *Lyomyceslancangjiangensis* Q. Li & C.L. Zhao, and *L.yunnanensis* C.L. Zhao by ellipsoid basidiospores. However, *L.lancangjiangensis* differs from *L.albofarinaceus* due to its membranaceous hymenial surface and narrower basidia (13.0–23.0 × 3.0–4.5 µm; Li et al. 2024b). The taxon *L.incanus* differs from *L.albofarinaceus* by its furfuraceous hymenial surface and narrower basidiospores (5.0–6.5 × 4.0–5.0 µm; Dong et al. 2024). The species *L.yunnanensis* differs from *L.albofarinaceus* by its grandinioid hymenial surface and narrower basidiospores (5.0–7.0 × 3.0–4.5 µm; Guan et al. 2023).

Morphologically, *Lyomycesalbomarginatus* resembles *L.lincangensis* J.H. Dong & C.L. Zhao, *L.luteoalbus* J.H. Dong & C.L. Zhao and *L.sinensis* J.H. Dong & C.L. Zhao by ellipsoid basidiospores. However, *L.lincangensis* differs from *L.albomarginatus* due to its coriaceous hymenial surface and wider basidiospores (4.5–6.5 × 3.5–5.0 µm; Dong et al. 2024). *L.luteoalbus* differs from *L.albomarginatus* due to its membranaceous hymenial surface and shorter tapering cystidia (12.0–17.0 × 2.0–3.5 µm; Dong et al. 2024). *L.sinensis* differs from *L.albomarginatus* due to its coriaceous hymenial surface and wider basidiospores (4.5–6.0 × 3.5–4.5 µm; Dong et al. 2024).

Morphologically, *Tubulicrinisalbobadius* resembles *T.hirtellus* (Bourd. & Galz.) John Erikss, *T.orientalis* Parmasto and *T.xantha* C.L. Zhao by cylindrical basidiospores. However, *T.hirtellus* differs from *T.albobadius* due to its porulose hymenial surface and longer basidiospores (7.0–8.5 × 2.0–2.5 µm; Hjortstam et al. 1988). The species *T.orientalis* differs from *T.albobadius* due to its hispidulous hymenial surface and narrower basidia (11.0–16.0 × 3.5–4.0 µm; Maekawa and Nordén 2022). The species *T.xantha* differs from *T.albobadius* by its furfuraceous hymenial surface and longer and narrower lyocystidia (78.0–192.5 × 5.8–7.5 µm; He et al. 2020).

Morphologically, *Xylodonmusicola* resembles *X.cremeoparinaceus* Q. Yuan & C.L. Zhao, *X.luteodontioides* Q. Yuan & C.L. Zhao and *X.wumengshanensis* Q. Yuan & C.L. Zhao by ellipsoid basidiospores. However, *X.cremeoparinaceus* differs from *X.musicola* due to its farinaceous hymenial surface and narrower basidiospores (3.5–4.5 × 2.5–3.5 µm; Yuan and Zhao 2024). The species *X.luteodontioides* differs from *X.musicola* due to narrower basidiospores (3.5–4.5 × 2.5–3.5 µm) and by possessing schizopapillate cystidia (29.5–37.0 × 2.5–3.5 µm; Yuan and Zhao 2024). The species *X.wumengshanensis* differs from *X.musicola* due to its bigger basidia (22.5–33.0 × 5.0–5.5 µm) and by possessing fusoid cystidia (14.5–22.0 × 5.5–6.5 µm; Yuan and Zhao 2024).

This discovery of five new species *viz. Hymenochaeteweishanensis*, *Lyomycesalbofarinaceus*, *L.albomarginatus*, *Tubulicrinisalbobadius*, and *Xylodonmusicola* enrich our knowledge of fungal diversity in the order Hymenochaetales. We anticipate that more undescribed taxa will be discovered throughout China after extensive collection combined with morphological and molecular analyses.

## Supplementary Material

XML Treatment for Hymenochaete weishanensis

XML Treatment for Lyomyces albofarinaceus

XML Treatment for Lyomyces albomarginatus

XML Treatment for Tubulicrinis albobadius

XML Treatment for Xylodon musicola

## ﻿Additional information

### Author ORCIDs

Yunfei Dai https://orcid.org/0009-0007-7734-4142

Qi Yuan https://orcid.org/0000-0002-6732-1656

Xin Yang https://orcid.org/0009-0009-7755-0581

Rui Liu https://orcid.org/0009-0009-0447-7766

Haisheng Yuan https://orcid.org/0000-0001-7056-140X

Changlin Zhao https://orcid.org/0000-0002-8668-1075

### Data availability

All of the data that support the findings of this study are available in the main text.
